# Multidisciplinary approach to COVID-19 risk communication: a framework and tool for individual and regional risk assessment

**DOI:** 10.1038/s41598-020-78779-0

**Published:** 2020-12-10

**Authors:** Rishi Ram Parajuli, Bhogendra Mishra, Amrit Banstola, Bhoj Raj Ghimire, Shobha Poudel, Kusum Sharma, Sameer Mani Dixit, Sunil Kumar Sah, Padam Simkhada, Edwin van Teijlingen

**Affiliations:** 1 Department of Civil Engineering, University of Bristol, Bristol, UK; 2 Science Hub, Kathmandu, Nepal; 3 Faculty of Health and Applied Sciences, University of the West of England, Bristol, UK; 4 Department of Research, Public Health Perspective Nepal, Pokhara, Nepal; 5 Faculty of Science, Health, and Technology, Nepal Open University, Lalitpur, Nepal; 6 Center for Molecular Dynamics Nepal, Kathmandu, Nepal; 7 Mid Yorkshire Hospitals NHS Trust, Wakefield, UK; 8 School of Human and Health Sciences, University of Huddersfield, Huddersfield, UK; 9 Faculty of Health and Social Sciences, Bournemouth University, Bournemouth, UK

**Keywords:** Natural hazards, Risk factors

## Abstract

The COVID-19 pandemic has exceeded over sixty-five million cases globally. Different approaches are followed to mitigate its impact and reduce its spreading in different countries, but limiting mobility and exposure have been de-facto precautions to reduce transmission. However, a full lockdown cannot be sustained for a prolonged period. An evidence-based, multidisciplinary approach on risk zoning, personal and transmission risk assessment in near real-time, and risk communication would support the optimized decisions to minimize the impact of coronavirus on our lives. This paper presents a framework to assess the individual and regional risk of COVID-19 along with risk communication tools and mechanisms. Relative risk scores on a scale of 100 represent the integrated risk of influential factors. The personal risk model incorporates age, exposure history, symptoms, local risk and existing health condition, whereas regional risk is computed through the actual cases of COVID-19, public health risk factors, socioeconomic condition of the region, and immigration statistics. A web application tool (http://www.covira.info) has been developed, where anyone can assess their risk and find the guided information links primarily for Nepal. This study provides regional risk for Nepal, but the framework is scalable across the world. However, personal risk can be assessed immediately from anywhere.

## Introduction

The coronavirus disease (COVID-19) outbreak originating in China in late 2019 has spread worldwide claiming 1/2 a million lives^1,2^. The rapid rate of human to human transmission of the virus has threatened the health and livelihood of the entire world^3^. The World Health Organization (WHO) declared it a pandemic on 11th March 2020^1^. Containment and quarantine of the virus contraction have been the major tool to control the spreading in early stage, however, the increasing rate of infections shown the limitations of this approach^4^. Many countries enforced strict lockdown to limit the spread of COVID-19, such as Italy and Spain, or during the initial phase of the outbreak as in Nepal^5^. WHO issued guidelines for responding to viruses prioritizing actions including maintaining hospital facilities, raising public awareness, and stocking up medical supplies^6^.

Public awareness of causative factors of COVID-19, its intensity, risk level, and consequences could help motivate people to adopt the required public health measures rather than ignoring or over-reacting during this pandemic. A preliminary study from China showed several psychological consequences arising during the COVID-19 pandemic^7^. Appropriate risk perception and communication could greatly help in reducing fear and increasing knowledge sharing during pandemic^8,9^. To predict the most likely scenarios, as governments and as individuals, the right tools need to be available.

Institutional and individual behaviour in society greatly affects the spread of infectious diseases^10^. Containment, contact tracing, and testing of infectious people should be priorities in the early stages of the pandemic^11^. Several low-income countries (LICs) including Nepal introduced strict lockdown measures during the early phase of the pandemic, but still failed to control the spreading of the virus. Even after a 2½ months-long of lockdown new infections are mounting up^12^. Citizen and institutional/governance awareness is always a key component in disaster risk reduction, something which is often lacking in LIC^13^.

Limiting economic activities to combat the pandemic have multi-dimensional impacts; increasing economic uncertainties and exacerbating the vulnerability of the improvised community^14^. Demand and supply chains all over the world have been disrupted^15^. United Nations (UN) agencies have reported that lockdown in some LIC resulted in hunger and poverty^16,17^. There needs to be a balance between controlling COVID-19 and maintaining economic activity including the supply of food and essentials though it is challenging^18^.

A personal and spatial risk assessment is required to cope with increasing challenges of the absolute lockdown. It paves the way to open the essential operations in the low-risk area by providing safety guidelines about the precaution measures that could fuel the socio-economy of the local community. Personal and regional risk assessment is a systematic and scientific way, in near-real-time (daily basis) to guide individuals and communities to take the appropriate decision. This is more effective in the countries like Nepal, where the current transmission pattern of COVID-19 is sporadic as early COVID-19 cases required the virus abroad with subsequent local transmission. Regular personalized risk indicator and spatial risk zonation not only provides up-to-date information but also can change risk perception and consecutively behaviour which could effectively be used to ease the strict lockdown measures in badly hit countries ^19,20^.

In this paper, we propose a framework for the COVID-19 risk assessment by incorporating the COVID-19 cases, exposure, immigration (quarantined data), public health facility, and population density, to access the regional and personal risk. We developed a near real-time COVID-19 Risk Assessment (COVIRA) tool based on the proposed framework. The COVIRA incorporates the virus transmission rate, public health risk, and population vulnerability, socio-economic status of the region, and importance of the region in overall context for essentials production and supply. Additionally, personal risk assessment provides individual vulnerability and risk of COVID-19 infection. Personalized risk communication could support limiting the spread of the virus and ultimately provide a better way to exit the pandemic. As, the personal, and regional risk computed through COVIRA reflect the recent scenario, an appropriate measure can be taken to remain safe from the infection and bring the life of people to normal as soon as possible.

## Result

The COVIRA is a dynamic system for regional and personal COVID-19 risk assessment based on the most recent data available. Results are presented in three segments; personal risk, regional risk assessment, and zonal importance to provide the overall scenario of COVID-19 risk in real-time. Personal risk provides the individual vulnerability towards this pandemic which will notify the risk of (i) an individual getting infected, (ii) COVID-19 infection and (iii) being infected through regional risk. Regional risk assessment provides COVID-19 transmission risk in the region, public health risk, socioeconomic risk and the overall risk. One major aspect of managing exit from the pandemic is prioritizing essential services by providing the regional importance maps and guidance. This would effectively be updated to guide the community and individual when the government policy changes for the priorities during different stages of a pandemic. All risk factors are fitted into a scale of 0 to 100 in the calculation.

### Personal risk assessment

Age and existing health conditions are two major factors contributing to COVID-19 risk. Data analysis from six countries (China, Italy, Spain, Germany, UK [United Kingdom] and USA [United States of America]) provide a fair relationship of these factors in patients’ death. Age is exponentially correlated with risk where underlying health conditions are contributing nearly 93% of overall death in UK and USA. Data suggest that older people are at high risk from COVID-19. A fraction of deaths to total deaths in each age group and percentage of non-survivors to total positive cases in corresponding age groups, both indicate an almost similar trend, where death percentage of total positive cases promisingly shows the exponential relationship with age as shown in Eq. (1). Data correlation between each country is shown in methodology with more details on supplementary materials from both perspectives.

1 $$y = \alpha e^{\beta x}$$

where, ‘y’ is a normalized risk factor; ‘x’ is the age in years (up to 90 years), normalized by mean 45.11 and standard deviation 27.32; α and β are coefficients having values (95% confidence level) 8.947 (5.955, 11.94) and 1.492 (1.272, 1.712) respectively; R-squared value of this relationship is 0.9538.

Underlying health condition from China^21,22^, the USA^23^ and the UK^24^ are also analyzed to find the correlation of different underlying health conditions over the age due to COVID-19. Guan et al.^21^ reported total cases and severe cases, where Zhou et al.^22^ reported total cases and corresponding deaths associated with different underlying health conditions. Garg et al.^23^ reported only hospitalization rates of several underlying health conditions in different age groups. The UK data published by Docherty et al.^24^ reported a total of 16,749 patients with different symptoms and underlying health conditions. Average of relative risk factors of different existing health conditions, comorbidities’ risk factor (CRF) from available data are provided in Table 1, detailed data can be found in supplementary materials.

**Table 1:** Relative risk of existing health condition to COVID-19.

Comorbidities	CRF
Asthma/chronic obstructive pulmonary disease	100
Cardiovascular/coronary heart disease	99
Renal disease	90
Diabetes	69
Hypertension	54
Obesity	54
Cancer	54
Cerebrovascular disease	48

**Figure 1:**
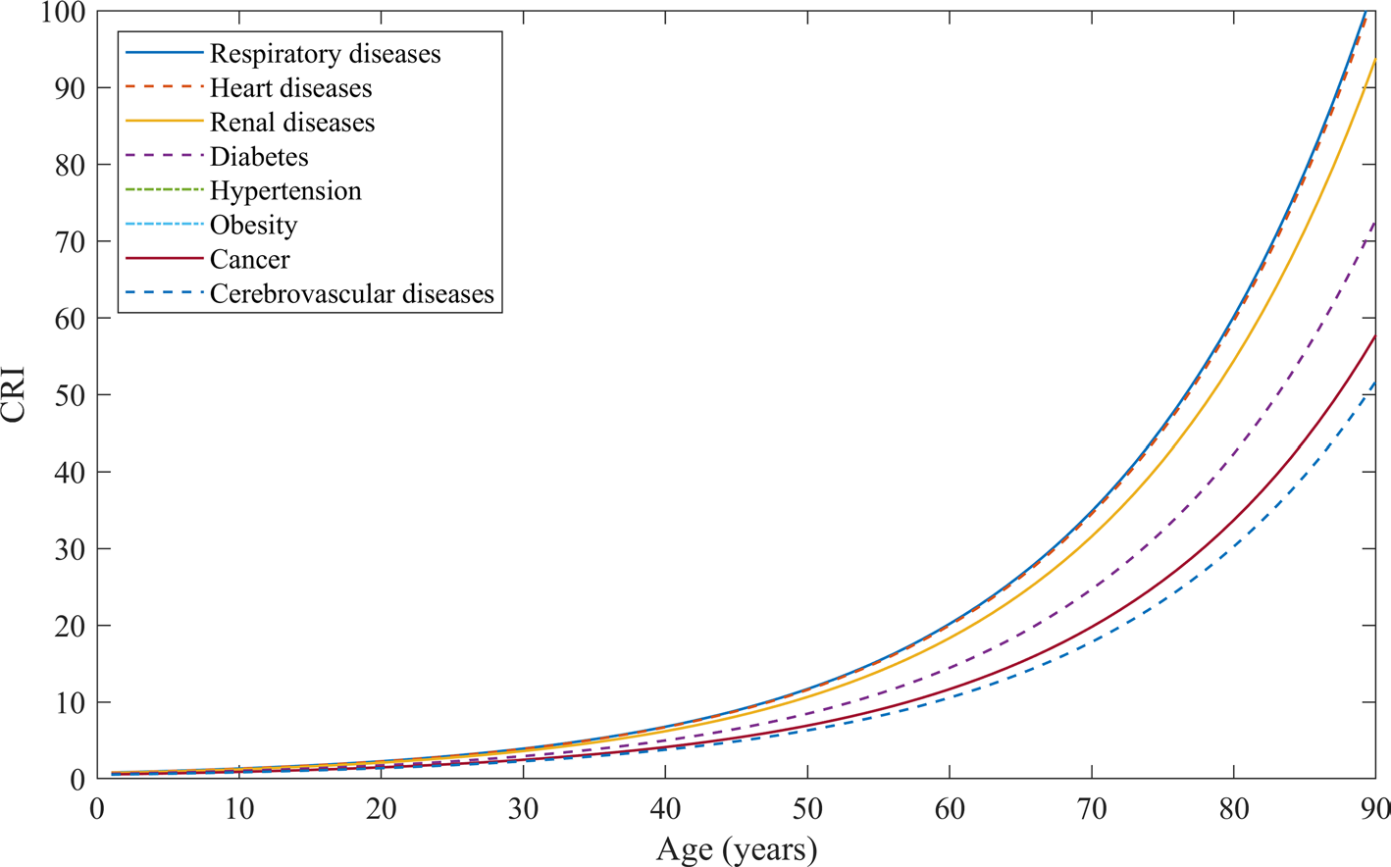
COVID risk index for men in relation with age and different comorbidities.

Following the trend of percentage of deaths with an existing health condition to the different age groups, another function (z) for a coefficient of comorbidity has been derived from UK data^25^ as shown in Eq. (2). Equation (3) provides total relative risk evaluation, COVID risk index (CRI) of an individual. Figure 1 shows the risk curves of people with age for different comorbidities in a normalized scale of 100.

2 $$z = ax^{2} + bx + c$$

where, coefficient values (with 95% confidence level) are, a = − 3.40646579722815 × 10E − 5 (− 4.477e−05, − 2.336e−05), b = 0.00672965343817772 (0.00563, 0.007829), and c = 0.635624565570039 (0.6123, 0.6589); with R-square value of 0.9994.

3 $$CRI\;(\% ) = y*\left[ {(1 - z) + z \times CRF/100} \right]$$

Risk is communicated as a five-point Likert scale to express very high, high, moderate, low and very low risk for an individual. Stratification of risk into five ranges is based on the distribution of the area under the risk curve. Where risk under the curve with maximum risk is distributed equally. Table 2 shows the range of CRI to represent a different level of risk. The area under curve of each range is depicted in the supplementary materials, Figure A-1.

**Table 2:**
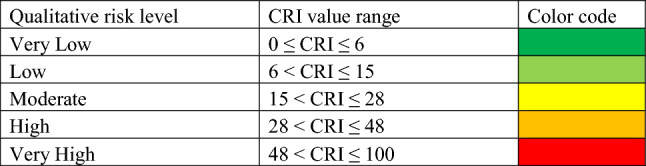
Qualitative representation of risk from CRI values.

### Probability of COVID infection for individual

Probability of COVID-19 infection has been assessed using exposure and local risk. A detail explanation to calculate the exposure is discussed in the methodology. Probability of infection has been shown in a five risk-levels on a linear scale (0–20–40–60–80–100). Assessing the symptoms of the patients reported in recent studies^21–23^, risk of having a positive case of COVID-19 in an individual is evaluated through their response to the questionnaire in a web app. Fever, cough and shortness of breath are major symptoms found in patients. Symptoms are only used to inform the respondent to contact to the nearest health care facilities depending on the level of their risks associated with symptoms.

### Regional risk assessment

**Figure 2:**
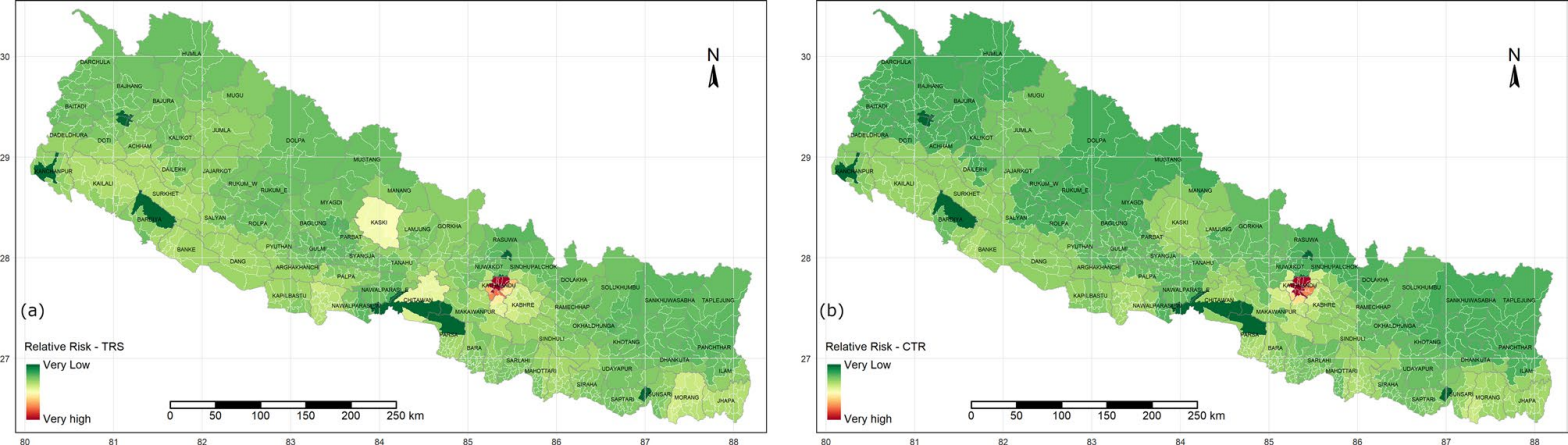
Relative risk maps of overall risk scenario, overall risk score of Nepal (**a**) and COVID-19 transmission risk (CTR) (**b**) prior to pandemic. Maps were produced from R programming version 3.5.3; source: https://cran.r-project.org/bin/windows/base/old/3.5.3/.

**Figure 3:**
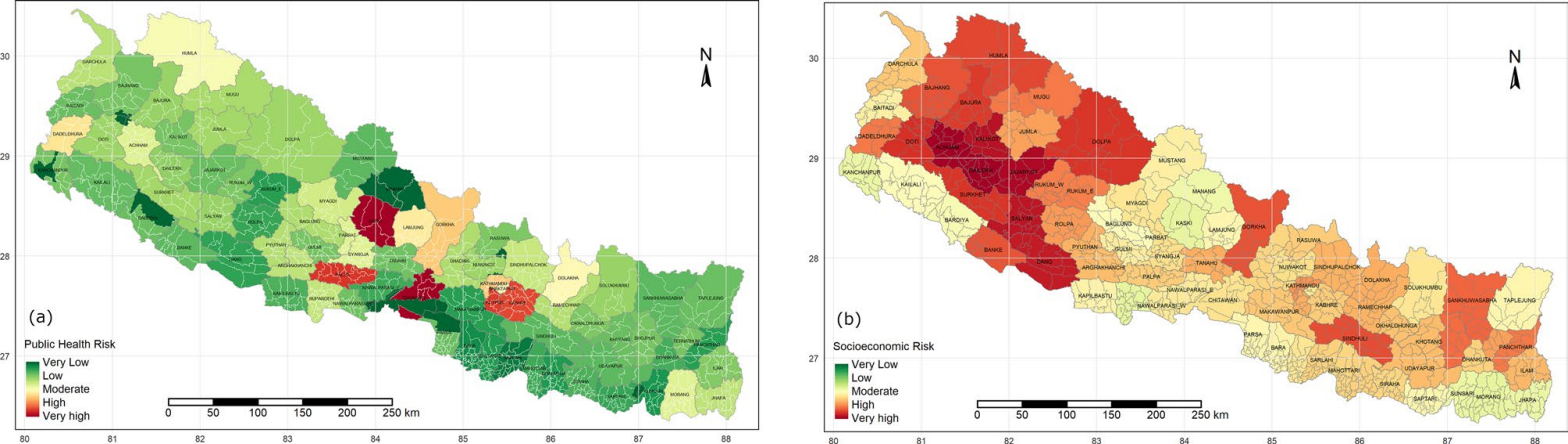
Public health risk (**a**) and socioeconomic risk (**b**) of Nepal for COVID-19 pandemic. Maps were produced from R programming version 3.5.3; source: https://cran.r-project.org/bin/windows/base/old/3.5.3/.

Risk zones have been mapped through a multidisciplinary approach to evaluate the regional risk, which is applicable for any region across the world. Figure 2 shows the relative risk maps of Nepal for overall risk of COVID-19 and the transmission risk of COVID-19 on the pre-pandemic state, the corresponding dataset are presented in Table A-9, supplementary materials. Public health risk, considering the underlying health condition and health service facilities in respective of the total population of that area are mapped along with socioeconomic risk level associated with COVID-19 as shown in Fig. 3.

**Figure 4:**
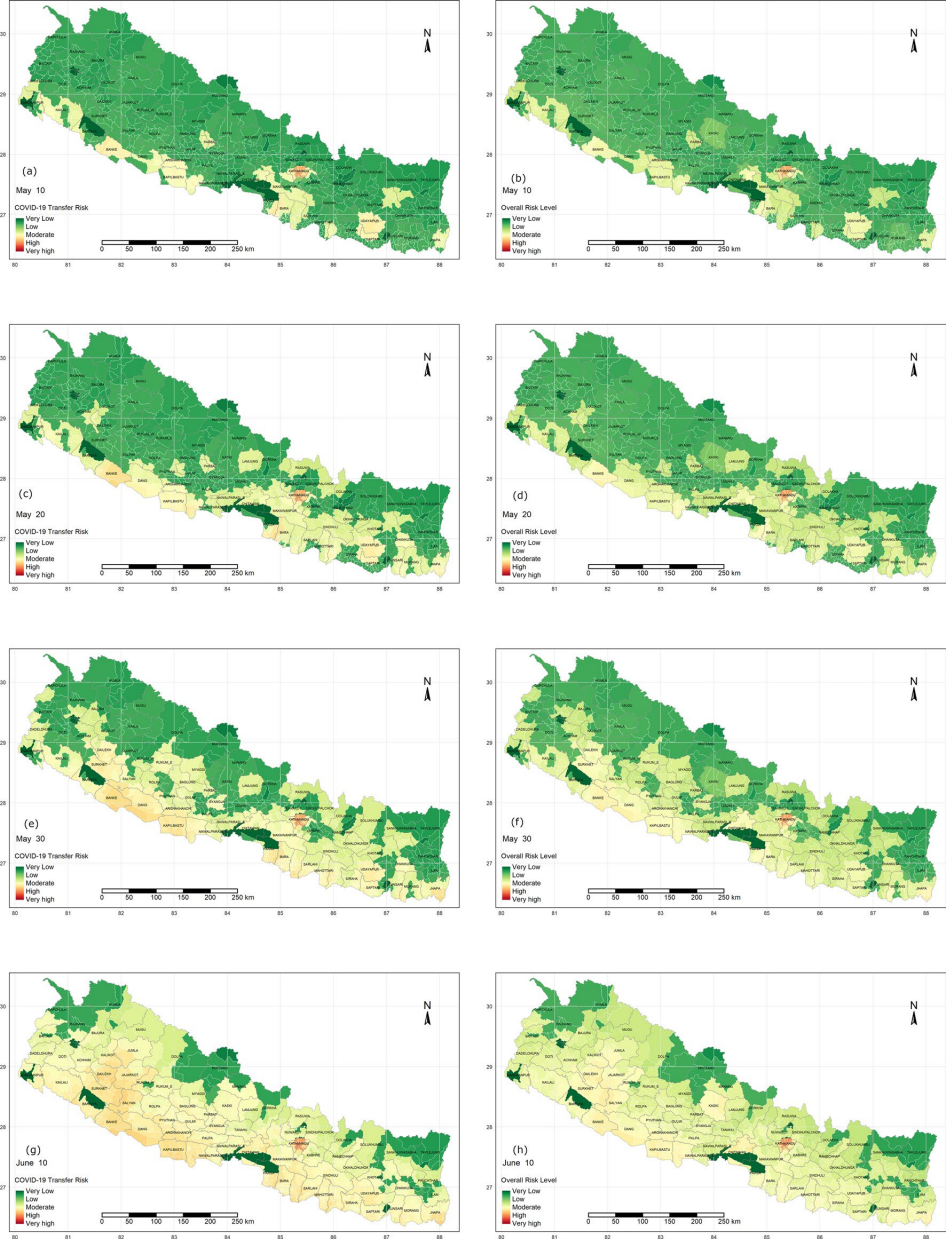
COVID 19 overall risk (right column) and CTR (left column), updated after the positive cases confirmed May 10, 20 and 30 and June 10 from top to bottom. Maps were produced from R programming version 3.5.3; source: https://cran.r-project.org/bin/windows/base/old/3.5.3/.

**Figure 5:**
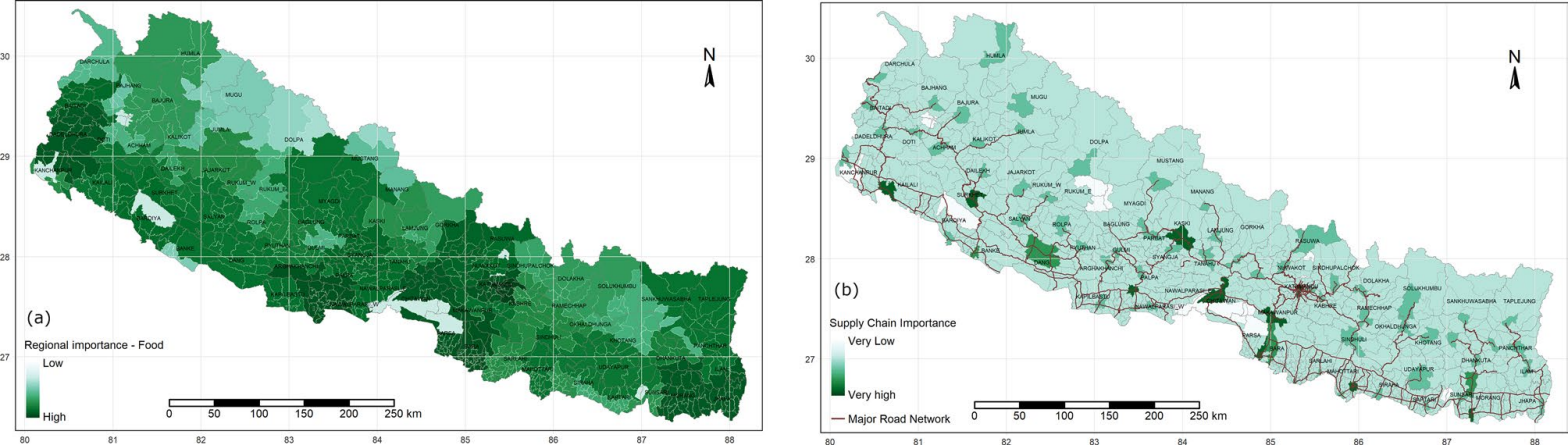
Regional importance food production (**a**) and supply-chain importance (**b**); maps were produced from R programming version 3.5.3; source: https://cran.r-project.org/bin/windows/base/old/3.5.3/.

The overall scenario of COVID-19 risk and transmission risk throughout the country, considering the influential factors are presented for the pre-pandemic scenario. Kathmandu is in the high-risk zone for both transmission and overall risk, however, most of the other areas with higher transmission risk are not associated with higher overall risk. Population exposure in Kathmandu is also highest in the country, hence the government should prioritize and localize the measures in the capital. CTR in southern boundaries is higher as it shares an open border with India, other areas with highly dense population, having more locations of exposure such as hotels and airports are in the upper side of the table. Figure 4(a–h) shows the timeline of CTR and overall risk across the country on May 10th, 20th, 30th and June 10th respectively. This can be updated regularly, upon receiving the official data. It is presented in a continuous scale range from very high to very low-risk zones. Regional importance zonation is mainly based on food (rice and maize) production and supply chain across the region, Fig. 5 shows the regional importance map of Nepal.

### COVIRA for risk assessment and communication

**Figure 6:**
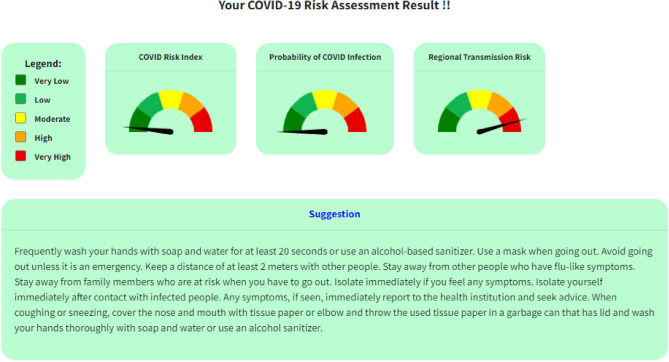
Result of COVIRA tool on-screen after personal risk assessment.

COVIRA (http://www.covira.info) was launched by the Nepal Engineers Association on 21st June 2020. Results are based on data provided by the user questionnaire, designed for personal risk assessment. Address, age, existing health condition, any current symptoms, exposure to infected persons were collected. Data entered by an individual through multiple checkboxes are converted into scores which are then used to calculate risk using the equations above. Personal risk values solely depend on the data provided by the individual, but regional risk assessment, CTR will be the result of the risk in their locality. Result will be provided on the web as shown in Fig. 6. Personalized messages are displayed in brief on information about risk and mitigation measures. Users can find the local risk in selected municipalities through the navigation on the page.

## Discussion

As the pandemic is spreading over the world, new symptoms and correlations are being unfolded. As per Radanliev et al.^26^ obesity, exercises, smoking are closely correlated to COVID-19. The COVIRA framework could unfold the risk-level of the person that would pave the way to move forward by minimizing the risk with no compromise in public health. Results from the model showed good resemblance of COVID-19 transmission risk when compared with no cases (Fig. 2a) and transmission risk on June 10th with cases over the country (Fig. 4g). Hence, the cases are arising in the region where relative risks are higher and spreading over the lower-risk zones.

For regions with very low risk, where no active cases have been found, a vigilance placed at the community level can cautiously ease life from the nationwide lockdown. Regional importance and zoning of several factors to sustain the minimum level of public life for the future, such as food and other essentials production and supply, would guide the community to act responsibly. The use of this tool will also greatly assist in easing tight lockdowns in places where mass testing is not available. A risk assessment using the tool will show those vulnerable in the region. It can also facilitate the entry of individuals into new zones after mobility is restored in countries, based on knowledge of population at risk.

The COVIRA currently developed for Nepal generates both personal and zonal risk with a current scenario for adequate measurement and this tool can be scaled up worldwide. The two indicators for personal risk reflect the chance of transmission, infection, and recovery under the stipulated environmental and health condition. If the risk of transmission is high, one can limit one’s interactions with other people, which reduces the exposure. However, if one has likely to have a low life-threatening risk even if anyone gets infected with the COVID-19 they can continue their regular job with necessary precautions. This could help the people remain alert based on their risk factors. The system generates the individual risk level along with public health advice to remain safe (see Fig. 6). This way, a community-level risk is also possible to get once a large number of people participate in this system.

Quantitative risk assessment is a challenging process, but a good method for communicating and making people aware of their vulnerability and risk due to COVID-19^27^. There is always a risk of entering the wrong information in a crowd-sourced data collection tool. However, if someone enters the wrong information and completes the assessment, it would only affect their own risk score since regional risk assessment relies on the depth of data available at community level.

The regional/zonal vulnerability risk is dynamic as the input variables are not static as these include, among others, the number of immigrants or COVID-19 positive cases. The COVIRA updates the vulnerability condition as the input parameters change. Figure 2a represents the base vulnerability risk map, (pre-COVID-19), and Fig. 4g is the latest vulnerability map based on the updated COVID infected cases and a number of immigrants quarantined from abroad. Specifically, a large number of people arrived from India after lockdown started and they are the majority of newly identified COVID-19 cases in this population. The base vulnerability map depicts that the urban area/region with international connections and regions bordering India have the highest base risk. And the total number of infected people has increased significantly in these high-risk regions in the base risk map^28^.

A recent study in Nepal shows “three in every five employees lost their jobs due to the COVID-19”, and the resulting hardship could be even worst next year if nationwide lockdown is continued^29^. Therefore, the personal risk and zonal risk map can help some people to continue their daily activities, based on its priority and risks. On March 24, when the Nepal government imposed the nationwide lockdown, there was only one active COVID-19 patient and no casualty. Instead of taking a local approach, the government imposed a nationwide absolute lockdown as its blanket approach, perhaps a local approach of containing the virus could have implemented for a better balance between health and socio-economic consequences.

The scenario could change rapidly based on the infected condition, local activity, and the immigration condition, data availability on time is always an issue. The reference data are taken from the high-income countries (Italy, Spain, Germany, USA, UK) and China which are different from LICs countries like Nepal. However, the range of data sets of those countries represents the risk scenario well across the world.

## Methodology

Risk assessment is principally based on the available data, risk assessment in pandemics relies on daily updates on infected cases and recoveries. Main three sources of data are used: (1) recent updates about COVID-19 (to find the relationships between potentially influential factors and to update the risk map); (2) demography of health condition and facilities, socioeconomic and other static data; and (3) questionnaire response in the app of an individual to assess the personal risk.

### Data sources

Published data related to COVID-19 cases from China^30^ (n = 1023), Italy^30^ (n = 1624), Spain^31^ (n = 16,680), Germany^32^ (n = 6512), the USA^33^ (n = 12,998) and the UK^25^ (n = 20,483) are used to establish the relationships of different factors associated with the virus spreading and loss of life. The individual risk depends upon age and underlying health conditions, however, other contextual demographic classifications, as like ethnicity may further extend in different countries. Regional risk assessment for most of the pandemic depends on population with an underlying health condition, health infrastructure facilities and services, water sanitation and hygiene status, poverty, literacy, population density, etc^34^. This study provides an example of regional risk assessment for Nepal, using the data published in national reports^35–37^. Daily updates on COVID cases and quarantines are linked with the Ministry of Health and Population web^12^. The political boundary of Nepal is obtained from the Survey Department, Nepal^38^ .

### Personal risk assessment

Personal risk assessment consists of major three parts: (1) individual vulnerability (danger to life when infected with COVID-19); (2) risk of infection if symptoms exist; and (3) risk of transmission to individuals are being evaluated from their questionnaire responses.

**Figure 7:**
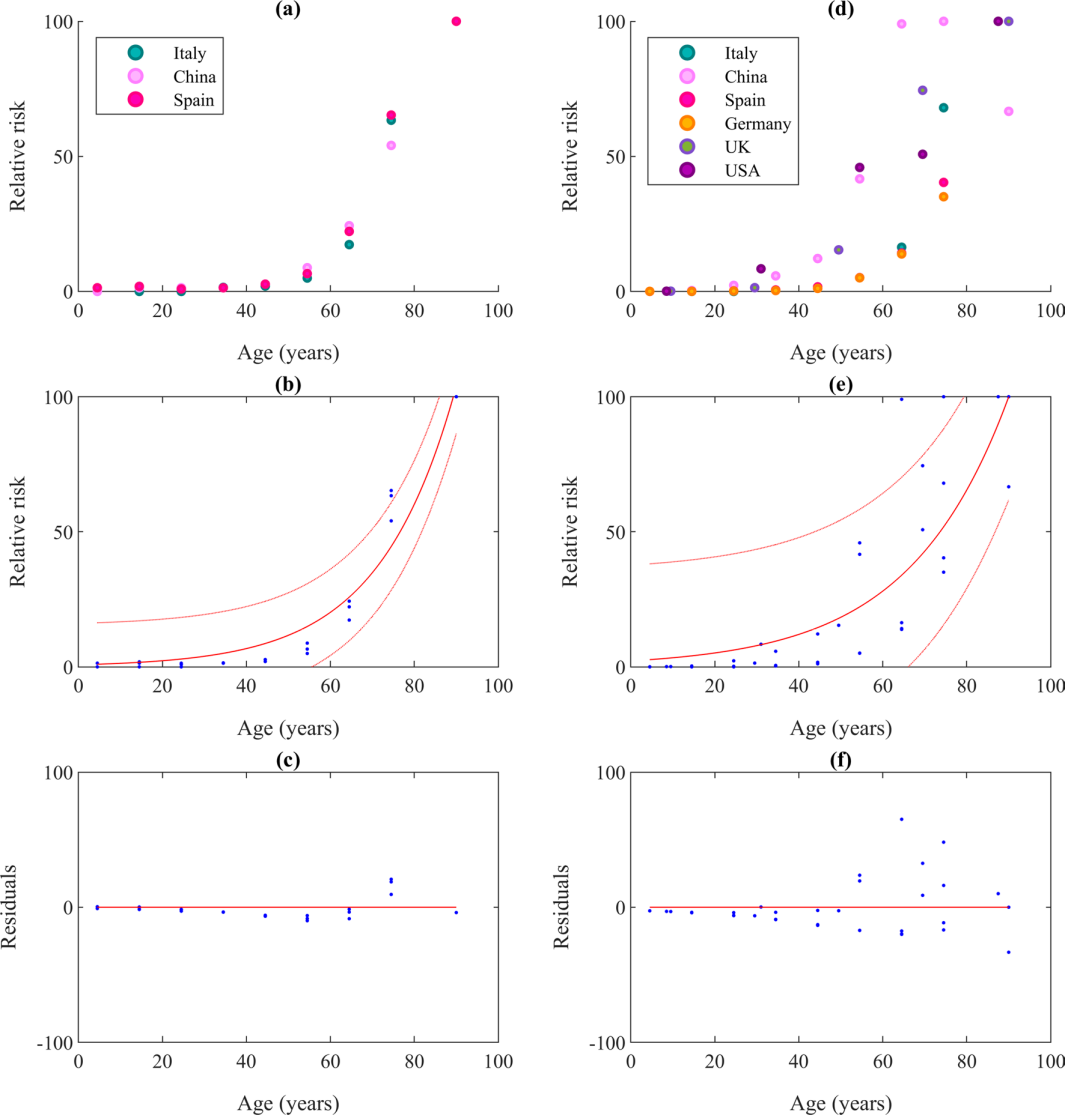
Correlation of recorded deaths and age: (**a**) normalized fatality percentages of cases of respective age group; (**b**) exponential curve fit to data of ‘a’ with 95% confidence boundaries; (**c**) corresponding residuals; (**d**) normalized fatality percentage of total fatality reported; (**e**) exponential curve fit to data of ‘d’ with 95% confidence boundaries; and (**f**) corresponding residuals.

Age and existing health conditions are two key factors for individual vulnerability. Distribution of death of different age groups is further converted in relative scale from different countries. Death toll distribution over the age group from the total death in the country and corresponding positive COVID cases of the age group are analyzed. Figure 7 shows the normalized fatality rates for different age groups from two perspectives; first column (a, b and c) shows the fatality rates in reported positive cases for each age group where the second (d, e and f) represent relative death percentage of each age group in totality.

Interestingly, relative death percentages of age group to the total death and relative death percentage to the confirmed cases of the corresponding age group show a very good correlation irrespective of sample size and country except for China. Data from China show a lower percentage of death to total death in the highest age group, however, the ratio of death to a positive case is highest in that age group. Equation (1) has been derived from death to positive cases percentage in age groups to represent the COVID-19 risk factor.

Underlying health condition of the reported cases is also analyzed using the data reported from China^21,22^, the USA^23^, and the UK^24^. Four sets of data are used to find the relative risk of different existing health conditions, even though all data sets are not in a similar format, they support each other in away. The major risk factor was established from the percentage of death and or severe condition of patients with different underlying health conditions. Obesity has not been listed in data from China but the USA and UK both have large numbers of patients even though the death percentage is not mentioned but is in nearly the same range of hypertension. Hence, the obesity risk factor is assigned similar to hypertension. The chronology of data tables and calculations are provided in the annex. The average of relative risk factors of different existing health conditions is provided in Table 1. A recent study on gender effect on mortality rates due to COVID-19 suggested the higher mortality rate for men (70.3% vs 29.7%)^39^. Hence, having the risk factor for a male is 100, the maximum risk factor for female will be 42.24. Total risk evaluated will be multiplied by 0.4224 for female.

Individual vulnerability is now calculated through the weightage of age, gender and existing health conditions. Recent data from the USA^23^ and the UK^25^ (as of April 31) show the death percentages of COVID cases with and without underlying health conditions are 89.3% (n = 1482) and 94.8% (n = 19,740). Following the trend of the percentage of deaths with an existing health condition in different age groups, another function for the coefficient of comorbidity has been derived from UK data as previously shown in Eq. (2).

### Probability of COVID-19 infection for individual

Table 3 provides the relative percentage of patients having different symptoms from the data provided in referred studies. Fever is the most common symptom where cough is almost found in a similar number of patients, shortness of breath, fatigue, and sputum production are other major symptoms. Considering the asymptomatic cases of COVID-19, providing the probability of having COVID-19 result is challenging. Hence, if someone reports any one symptom or combination of different symptoms, recommendations will be displayed to contact the nearest health care facilities.

**Table 3:** Symptoms and corresponding relative percentages.

Symptoms	Relative percentage
Fever	100
Cough	87
Shortness of breath	57
Fatigue	34
Sputum production	31
Myalgia	24
Sore throat	18
Headache	17
Chest pain	17
Diarrhea	14
Nausea or vomiting	13
Chills	13
Nasal congestion	12

In addition, exposure of any individual can be calculated through their profession, daily activities, travel, and meeting with (a-symptomatic) COVID-19 infected people. Exposure and corresponding values are shown in the table in supplementary materials. Now, considering the exposure and regional risk, the total probability of COVID-19 infection risk will be calculated. In the initial cases in Nepal, as reported in media briefings by the Ministry of Health and Population, very few of the positive cases have symptoms. Hence, we proposed the risk of COVID-19 infection as a product of local risk and exposure. Equation (4) provides the probability of infection, where the upper bound value will be limited to 100, even if it exceeds.

4 $${\text{Probability of COVID - 19 infection}}\, = \,{\text{regional risk}} \times {\text{exposure}}$$

Exposure can be evaluated through the occupation, proximity to public and other persons and their day to day safety measures. Studies from Italy^40^, and six Asian countries^41^ suggested the risk levels for different occupational groups, where drivers and health workers have higher exposure and thus higher risk. Exposure level on a scale of 0–100 is provided in Tables 4 and 5 based on these recent studies.

**Table 4:** Occupation and exposure value.

Occupation	Exposure Value
Driver and other transport work	100
Medical doctor	20
Nurse and other health workers	67
Shops or supermarket salesperson	50
Domestic housekeeper/cleaner	50
Religious professional	40
Teacher/trainer/professor	40
Travel attendant, guide	33
Construction labour	33
Security/police/fire fighter	20
Receptionists/bar tenders	20
Agriculture	20
Others	10
None	0

**Table 5:** Proximity and exposure value.

Activities	Exposure value
Travelled to COVID-19 infected area recently	100
Met with known COVID19 infected person	100
Have to be in proximity of less than 2 m in the workplace (or in the field, as part of the job) with public	90
Need to travel/walk in public areas where maintaining the distance of more than 2 m is impossible	80
Need to attend meetings in person occasionally	70
Have to be in proximity of less than 2 m in the workplace with other colleagues	60
Have more than one family members, who need to go out and meet other people	50
Have a family member, who need to go out and meet other people	25
None	0

In addition, preventive measures including using of face masks, keeping physical distances, wearing sunglasses in daily life reduce exposure and hence risk^42^.A recent study defined physical distancing in relation to risk of COVID-19 infection in terms of occupancy, ventilation, type of group activity. Jones et al.^43^ presented the level of risk in different scenarios where occupancy and wearing mask have equal importance in reducing risk. Hence, contribution in the reduction of exposure has been introduced as shown in Table 6 based on results presented by Chu et al.^42^ and Jones et al.^43^. Considering both studies, wearing a face mask, regular sanitization and maintaining distance show a similar level of protection. Differences in risk presented due to each preventive measure are directly used to reduce the exposure level. Hence considering the minimum exposure level (50); with all preventive measures, net exposure is nullified.

**Table 6:** Preventive measures and deduction in exposure value.

Preventive measures	Exposure value
Wear a mask when going to crowded areas	− 14
Sanitize hands using hand washing or sanitizer	− 14
Maintain physical distance when going outside	− 14
Use sunglasses/face shields	− 8

### Regional risk assessment

A multidisciplinary framework to evaluate the regional risk has been proposed to provide support on risk zoning. COVID-19 risk can be stratified into different levels based on transmission risk, public health status and facilities and socioeconomic condition. Population with an underlying health condition and older people are more vulnerable^44^, hence those regions with different existing health condition population are assigned score in a scale of 100. Available health facilities like hospitals, primary health care centres, health posts and female community health volunteers are also relatively scored in a scale of 0–100, throughout the country, in a proportion of the population.

Populations with higher poverty levels are more vulnerable during the pandemic, loss of a job, shortage of food could push them further down in poverty, likely to have poor nutrition, and loosening the immunity power. Hence, the socio-economic index of the region has also contributed to the vulnerability of the region, where literacy rates and water, sanitation and hygiene indexes are also used to further clarify the risk scenario.

**Figure 8:**
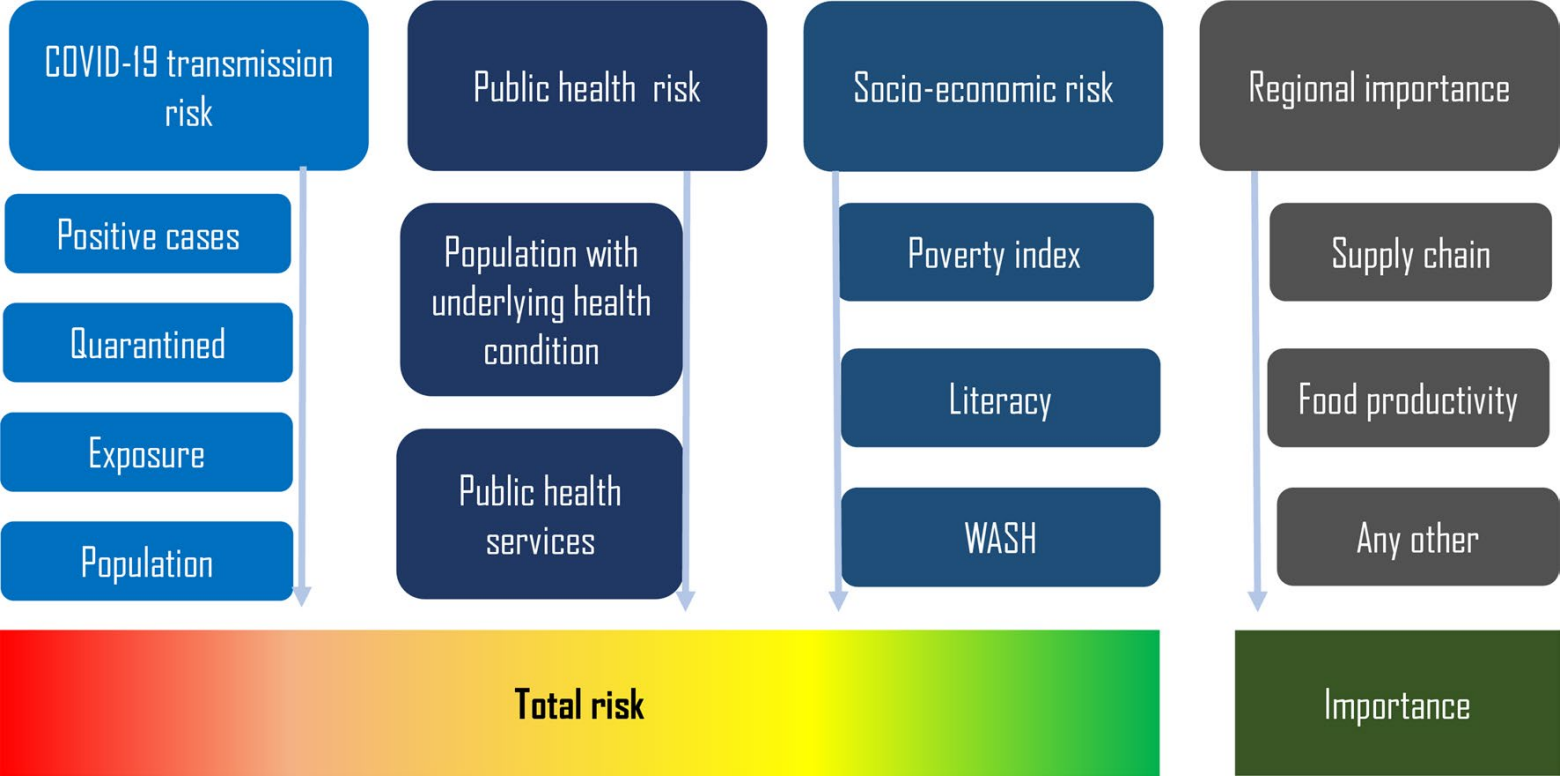
Framework and components for total COVID-19 risk evaluation.

Regional food production capacity and type, major supply chain hubs and network are further categorized with relative indices. Government guidelines and policies along the pandemic period may vary additional parameters to consider in importance zoning. General components of the framework are shown in Fig. 8. There is a lack of proper statistical data to calculate the factors for different components in the overall risk calculation related to this pandemic, hence, a Delphi method has been used to propose the equations^45,46^. All maps are produced through R programming that is available as Free Software Foundation’s GNU General Public License in source code form^38^.

#### COVID-19 transmission risk

Human-to-human transmission of the virus poses higher risk where more positive cases are found, other factors contributing to regional risk are, quarantined people in the region, who have visited or travelled from high-risk zones; exposure of the region to other regions of high risk and the population density. Equation 5 has been proposed for COVID-19 transmission risk.

5 $${\text{CTR}} = \left[ {0.6 \times {\text{PCS}}\, + \,0.1 \times {\text{QNT}}\, + \,0.1*{\text{EXP}}\, + \,0.2*{\text{POD}}} \right]$$

where, PCS = positive case score. QNT = quarantined people score. EXP = community exposure score. POD = population density score.

PCS is calculated in a relative scale of 0–100, considering the total active cases in and nearby region. Having zero cases, there will be no score of PCS but when the region witnessed any case then the risk score starts from 50. The reason behind the benchmark of ‘50’ is that, any active case may infect further cases in that community, hence assigned 50 for the first case, then risk will be increased in logarithmic scale to be 100 when cases will match with total household in the community. The score matrix of PCS for the region is calculated as shown in Eq. (6). The risk transmission possibility of the active cases from an administrative region to a nearby region is also considered. Most people from the boundary region could travel to the neighbouring region for shopping, work or trade when there is no restriction on movement. All adjacent regions of the index region having cases is considered the same active case to compute the PCS within the 10 km buffer. It is considered half in the administrative regions within another 10 km from the main region having active cases.

6 $${\text{PCS}} = 100 \, {-} \, \log \, ({\text{a/b}})*50/(\log (1/{\text{b}}))$$

where ‘a’ is the number of active cases in the region ‘b’ is a total number of households. Average household size of Nepal is 4.6^47^, which is used to calculate the household numbers in each administrative unit. The risk will be minimized over time if no cases are found in the considered administrative zone. Considering the characteristics of COVID-19 as reported by Lauer et al., time of probable exposure to the detection of the positive case is up to 60 days for most of the cases, however very few are longer but less than 90 days^48^. Hence, any administrative unit, considered in this study, having null positive cases for 60 consecutive days, PCS will be reduced to 50% of its value and after another consecutive 30 days (total 90 days) of no positive cases reports, PCS will be nullified.

The score for the quarantined people in the region follows the similar approach of the PCS. Exposure of the region is scored based on facilities those use by the people out of the region, like hotels, airports are considered. International arrivals include international airport and the other immigration points. Exposure score is tabulated in Table 7 for different facilities in the region.

**Table 7:** Exposure values for the combination of different facilities.

International arrivals	Domestic airport	3 + star hotels	Other hotels	EXP value
✓	✓	✓	✓	100
✓	×	×	✓	90
×	✓	✓	✓	80
×	✓	×	✓	70
×	×	✓	✓	60
×	×	×	✓	50

Population density of the region linearly correlates with the prevalence rate of infectious diseases^49^. Hence, the score for the population density of the region is relatively linearly distributed throughout the country with the minimum assigned score of 20.

#### Public health risk

People with a different underlying health condition (UHC) and the low availability of health facilities (HF) in the region are associated with the public health risk (PHR) of that region. People with different UHC in the region are scored in a single value with the weightage factors as of Table 1. Hospitals, primary health care centres, and health posts are assigned 100, 10, and 1 respectively for each unit. Summation of unit values in the region will be divided by the population in the region. Relative score on a scale of 100 will be calculated for each region. Both UHC and PHF are on the relative scale of 100. Hence, considering only 20% would potentially get service to recover from a critical stage, PHR is given by Eq. (7).

7 $${\text{PHR}} = {\text{UHC }}{-}{\text{ HF}}*0.2$$

Further assessment of public health in the community can be extended with different pandemic and post-disaster scenarios. Natural hazard and structural vulnerability, production and supply of medical facilities and medicines along with managerial and social engagement can also be considered in the public health vulnerability. For the scope of COVID-19, this study confined within the smaller boundary.

#### Socioeconomic risk

Socioeconomic risk (SER) for the pandemic is also considered in the integrated risk analysis. Poverty not only increases the risk of hunger, but poor people are also often not able to afford the cost of the required precautionary measures. Lower literacy rate indicates the lower level of awareness and a similarly lower index of water sanitation and hygiene indicates higher chances of spreading of the virus. Poverty index (PVI), literacy rate (LTR) and water, sanitation and hygiene index (WASH) of the region are relatively scored in a scale of 100. Awareness campaigns through media and community can increase the public awareness for even simply literate people so it will be given lower factor, comparison to poverty and WASH which would not change within a short time frame. Combining the factors, SER can be represented by Eq. (8).

8 $${\text{SER}} = 0.4*{\text{PVI}} + 0.2*{\text{LTR}} + 0.4*{\text{WASH}}$$

#### Regional importance

Food production and supply is the most important to maintain general health and well-being in the community, which ultimately support to fight the pandemic. Hence, major hubs of supply chain network (SCN) are mapped for their importance in the scale of 100 as shown in Table 8. Similarly, food productivity (FOOD) of the region is also relatively scored. Rice, wheat and maize production are taken into account with their planting and harvesting time which will be more crucial for that region. Additionally, the supply of fertilizers, seeds and other supporting materials should be available within the very short time frame. We cannot postpone the farming of such crops by months or even weeks, it depends on the climate and weather. Food production and supply chain, both are equally important, hence, regional importance (REG) can be represented in separate maps.

**Table 8:** Score of the major hub for the supply chain network.

Supply chain hubs	Score
Capital city	100
Provincial headquarters	90
Major cities/hubs other than above	70
District headquarters	50
Others	30

The overall risk will be calculated by combining CTR, PHR and SER, on the other hand, regional importance will be mapped. CTR is the major component in total risk score (TRS), where PHR and SER will further escalate the risk, as proposed in Eq. (9).

9 $${\text{TRS}}\, = \,{\text{CTR}}*(0.{6}*{\text{PHR}} + 0.{4}*{\text{SER}})/{1}00$$

## Conclusion

COVIRA, a risk assessment and communication tool has been designed to provide an overall risk assessment framework for COVID-19. The tool has been tested for Nepal but can be easily adapted across the globe since risk factors are very similar worldwide. The empirical models fitted through the available data sets of COVID-19 are used for evaluation of individual risk, considering major risk factors identified into the historical dataset. Personal risk assessment through questionnaire provides the individual risk and communicate the rules and guidelines for awareness. A regional risk assessment would support the government and community to act locally, which will give a clear way out to manage the risk wisely, refrained from any compromise on public health. Specifically, the regional risk and transmission map could help support essential supply chains at the time of absolute lockdown across the country. This multidisciplinary, holistic approach of risk assessment and communication can be extended to any other future pandemic and natural disasters.

## Supplementary Information

Supplementary information

## Data Availability

All data and models generated or used during the study appear in the submitted article including supplementary materials.
