# Supplementary material for: Multidisciplinary approach to COVID-19 risk communication: a framework and tool for individual and regional risk assessment
Source: Sci Rep. 2020 Dec 10;10:21650. doi: 10.1038/s41598-020-78779-0 (PMC7729931; doi:10.1038/s41598-020-78779-0)
Supplement: Supplementary file 1 — Supplementary information [file 41598_2020_78779_MOESM1_ESM.docx]

Multidisciplinary approach to COVID-19 risk communication: A framework and tool for individual and regional risk assessment

Rishi Ram Parajuli^1^, Bhogendra Mishra^2*^, Amrit Banstola^3,4^, Bhoj Raj Ghimire^2,5^, Shobha Poudel^2^, Kusum Sharma^2^, Sameer Mani Dixit^6^, Sunil Kumar Sah^7^, Padam Simkhada^8^ and Edwin van Teijlingen^9^

^1^Department of Civil Engineering, University of Bristol, UK

^2^Science Hub, Kathmandu, Nepal

^3^ Faculty of Health and Applied Sciences, University of the West of England, UK

^4^Department of Research, Public Health Perspective Nepal, Nepal

^5^Nepal Open University, Lalitpur, Nepal

^6^Center for Molecular Dynamics Nepal, Nepal

^7^Mid Yorkshire Hospitals NHS Trust, Leeds Teaching Hospital, UK

^8^ School of Human and Health Sciences, University of Huddersfield, UK

^9^Faculty of Health and Social Sciences, Bournemouth University, UK

*Corresponding author, Email: bmishra@sciencehub.org.np

### Data for proportion of death under each age group in total death

Table A-1 Death cases reported under each age group (Numbers)

| Age group | Country | | | | | | | |
| --- | --- | --- | --- | --- | --- | --- | --- | --- |
|  | China<sup>28</sup> | Italy^27^ | Spain^28^ | Germany^29^ | USA (NY)^30^ | | UK^24^ |  |
| 0-9 | 0 | 0 | 2 | 1 | 0-17 | 6 | 0-19 | 11 |
| 10-19 | 1 | 0 | 5 | 1 | 18-44 | 531 | 20-39 | 149 |
| 20-29 | 7 | 0 | 22 | 6 | 45-64 | 2908 | 40-59 | 1647 |
| 30-39 | 18 | 4 | 56 | 14 | 65-74 | 3217 | 60-79 | 7971 |
| 40-49 | 38 | 10 | 174 | 47 | 75-100 | 6336 | 80+ | 10705 |
| 50-59 | 130 | 43 | 525 | 211 |  | | | |
| 60-69 | 309 | 139 | 1459 | 580 |  |  |  |  |
| 70-79 | 312 | 578 | 4151 | 1467 |  |  |  |  |
| 80+ | 208 | 850 | 10286 | 4185 |  |  |  |  |

Table A-2 **Pearson's Correlations**

|  | | | | | | | | | | | |
| --- | --- | --- | --- | --- | --- | --- | --- | --- | --- | --- | --- |
| **Variable** | |  | | **China** | | **Italy** | | **Spain** | | **Germany** | |
| 1. China |  | Pearson's r |  | — |  |  |  |  |  |  |  |
|  |  | p-value |  | — |  |  |  |  |  |  |  |
|  |  | Upper 95% CI |  | — |  |  |  |  |  |  |  |
|  |  | Lower 95% CI |  | — |  |  |  |  |  |  |  |
| 2. Italy |  | Pearson's r |  | 0.672 |  | — |  |  |  |  |  |
|  |  | p-value |  | 0.047 |  | — |  |  |  |  |  |
|  |  | Upper 95% CI |  | 0.924 |  | — |  |  |  |  |  |
|  |  | Lower 95% CI |  | 0.014 |  | — |  |  |  |  |  |
| 3. Spain |  | Pearson's r |  | 0.571 |  | 0.970 |  | — |  |  |  |
|  |  | p-value |  | 0.109 |  | < .001 |  | — |  |  |  |
|  |  | Upper 95% CI |  | 0.895 |  | 0.994 |  | — |  |  |  |
|  |  | Lower 95% CI |  | -0.150 |  | 0.862 |  | — |  |  |  |
| 4. Germany |  | Pearson's r |  | 0.547 |  | 0.957 |  | 0.999 |  | — |  |
|  |  | p-value |  | 0.128 |  | < .001 |  | < .001 |  | — |  |
|  |  | Upper 95% CI |  | 0.888 |  | 0.991 |  | 1.000 |  | — |  |
|  |  | Lower 95% CI |  | -0.185 |  | 0.804 |  | 0.994 |  | — |  |
|  | | | | | | | | | | | |

Table A-3 Death cases reported under each age group (percentage)

| Age group | Country | | | | | | | |
| --- | --- | --- | --- | --- | --- | --- | --- | --- |
|  | China | Italy | Spain | Germany | USA (NY) | | UK | |
| 0-9 | 0.00% | 0.00% | 0.01% | 0.02% | 0-17 | 0.05% | 0-19 | 0.05% |
| 10-19 | 0.10% | 0.00% | 0.03% | 0.02% | 18-44 | 4.09% | 20-39 | 0.73% |
| 20-29 | 0.68% | 0.00% | 0.13% | 0.09% | 45-64 | 22.37% | 40-59 | 8.04% |
| 30-39 | 1.76% | 0.25% | 0.34% | 0.21% | 65-74 | 24.75% | 60-79 | 38.92% |
| 40-49 | 3.71% | 0.62% | 1.04% | 0.72% | 75-100 | 48.75% | 80+ | 52.26% |
| 50-59 | 12.71% | 2.65% | 3.15% | 3.24% |  | | | |
| 60-69 | 30.21% | 8.56% | 8.75% | 8.91% |  |  |  |  |
| 70-79 | 30.50% | 35.59% | 24.89% | 22.53% |  |  |  |  |
| 80+ | 20.33% | 52.34% | 61.67% | 64.27% |  |  |  |  |

### Data for proportion of death under each age group in total cases of that age group

Table A-4 Fatality percentage of confirmed cases under each age group

| Age group | Country | | |
| --- | --- | --- | --- |
|  | China | Italy | Spain |
| 0-9 | 0.00% | 0.00% | 0.29% |
| 10-19 | 0.20% | 0.00% | 0.39% |
| 20-29 | 0.20% | 0.00% | 0.19% |
| 30-39 | 0.20% | 0.30% | 0.28% |
| 40-49 | 0.40% | 0.40% | 0.57% |
| 50-59 | 1.30% | 1.00% | 1.39% |
| 60-69 | 3.60% | 3.50% | 4.69% |
| 70-79 | 8.00% | 12.80% | 13.79% |
| 80+ | 14.80% | 20.20% | 21.12% |

Table A-5 **Pearson's Correlations**

| **Variable** | |  | | **China** | | **Italy** | | **Spain** | |
| --- | --- | --- | --- | --- | --- | --- | --- | --- | --- |
| 1. China |  | Pearson's r |  | — |  |  |  |  |  |
|  |  | p-value |  | — |  |  |  |  |  |
|  |  | Upper 95% CI |  | — |  |  |  |  |  |
|  |  | Lower 95% CI |  | — |  |  |  |  |  |
| 2. Italy |  | Pearson's r |  | 0.993 |  | — |  |  |  |
|  |  | p-value |  | < .001 |  | — |  |  |  |
|  |  | Upper 95% CI |  | 0.999 |  | — |  |  |  |
|  |  | Lower 95% CI |  | 0.968 |  | — |  |  |  |
| 3. Spain |  | Pearson's r |  | 0.994 |  | 0.999 |  | — |  |
|  |  | p-value |  | < .001 |  | < .001 |  | — |  |
|  |  | Upper 95% CI |  | 0.999 |  | 1.000 |  | — |  |
|  |  | Lower 95% CI |  | 0.972 |  | 0.996 |  | — |  |
|  | | | | | | | | | |

## Calculation of health condition risk factor

Table A-6 Percentage of patients with different underlying health conditions

| Source | Guan et al^6^ | | Zhou et al^7^ | | Average of both data (in %) | Relative risk factor |
| --- | --- | --- | --- | --- | --- | --- |
| Comorbidities | Total cases | Severe cases | Total cases | Death cases |  |  |
| Chronic obstructive pulmonary disease | 12 | 6 | 6 | 4 | 56% | 100 |
| Diabetes | 81 | 28 | 36 | 17 | 38% | 69 |
| Hypertension | 165 | 41 | 58 | 26 | 30% | 54 |
| Cardiovascular/Coronary heart | 27 | 10 | 15 | 13 | 55% | 99 |
| Cerebrovascular | 15 | 4 |  |  | 27% | 48 |
| Cancer | 10 | 3 |  |  | 30% | 54 |
| Renal | 8 | 3 | 2 | 2 | 50% | 90 |

Table A-7 Death cases of UK, with and without existing health conditions (data of April 31)

| Age | with | without | Total | % with health condition |
| --- | --- | --- | --- | --- |
| 0-19 | 7 | 3 | 10 | 70% |
| 20-39 | 117 | 28 | 145 | 81% |
| 40-59 | 1401 | 184 | 1585 | 88% |
| 60-79 | 7270 | 433 | 7703 | 94% |
| 80+ | 9925 | 372 | 10297 | 96% |

Table A-8 Percentage of patients with different symptoms

| Source | Guan et al^20^ | Zhou et al^21^ | Garg et al^22^ |
| --- | --- | --- | --- |
| Country | China | China (Wuhan) | USA |
| Sample | 1099 | 191 | 180(Max) |
| Fever | 88.7 | 94 | 85 |
| Nasal congestion | 4.8 |  | 16.1 |
| Headache | 13.6 |  | 16.1 |
| cough | 67.8 | 79 | 86.1 |
| Sore throat | 13.9 |  | 17.8 |
| Sputum production | 33.7 | 23 |  |
| Fatigue | 38.1 | 23 |  |
| Shortness of breathe | 18.7 |  | 80 |
| Nausea or vomiting | 5 | 4 | 24.4 |
| Diarrhea | 3.8 | 5 | 26.7 |
| Myalgia | 14.9 | 15 | 34.4 |
| chills | 11.5 |  |  |
| Chest pain |  |  | 15 |

Table Table A- 9 CTR and TRS in the pre-Covid scenario and October 15 in Palikas of Nepal (the smallest administrative unit).

| **District** | **Palikas** | **Code** | **CTR Oct 15** | **TRS Oct 15** | **CTR precovid** | **TRS precovid** |
| --- | --- | --- | --- | --- | --- | --- |
| Taplejung | Phaktanglung | 10101 | 39.55 | 35.35 | 5.21 | 7.88 |
| Taplejung | Mikwakhola | 10102 | 41.96 | 37.28 | 5.21 | 7.88 |
| Taplejung | Meringden | 10103 | 50.92 | 44.45 | 5.21 | 7.88 |
| Taplejung | Maiwakhola | 10104 | 50.83 | 44.37 | 5.16 | 7.84 |
| Taplejung | Aathrai Tribeni | 10105 | 46.59 | 40.99 | 5.21 | 7.88 |
| Taplejung | Phungling | 10106 | 44.36 | 39.20 | 5.21 | 7.88 |
| Taplejung | Pathibhara Yangwarak | 10107 | 39.55 | 35.13 | 5.21 | 7.65 |
| Taplejung | Sirijangha | 10108 | 41.96 | 37.05 | 5.21 | 7.65 |
| Taplejung | Sidingba | 10109 | 39.68 | 35.23 | 5.15 | 7.60 |
| Sankhuwasabha | Bhotkhola | 10201 | 11.87 | 13.23 | 5.02 | 7.75 |
| Sankhuwasabha | Makalu | 10202 | 52.28 | 45.56 | 5.08 | 7.80 |
| Sankhuwasabha | Silichong | 10203 | 55.21 | 47.91 | 5.26 | 7.94 |
| Sankhuwasabha | Chichila | 10204 | 55.21 | 47.91 | 5.26 | 7.94 |
| Sankhuwasabha | Sabhapokhari | 10205 | 53.52 | 46.55 | 5.26 | 7.94 |
| Sankhuwasabha | Khandbari | 10206 | 57.54 | 49.77 | 5.26 | 7.94 |
| Sankhuwasabha | Panchakhapan | 10207 | 55.21 | 47.91 | 5.26 | 7.94 |
| Sankhuwasabha | Chainpur | 10208 | 55.19 | 47.89 | 5.26 | 7.94 |
| Sankhuwasabha | Madi | 10209 | 49.77 | 43.55 | 5.20 | 7.89 |
| Sankhuwasabha | Dharmadevi | 10210 | 52.99 | 46.13 | 5.20 | 7.89 |
| Solukhumbu | Khumbupasanglahmu | 10301 | 46.11 | 40.62 | 5.12 | 7.82 |
| Solukhumbu | Mahakulung | 10302 | 50.65 | 44.25 | 5.09 | 7.80 |
| Solukhumbu | Sotang | 10303 | 50.79 | 44.36 | 5.14 | 7.84 |
| Solukhumbu | Dudhkoshi | 10304 | 46.78 | 41.15 | 5.14 | 7.84 |
| Solukhumbu | Thulung Dudhkoshi | 10305 | 45.51 | 39.82 | 5.17 | 7.55 |
| Solukhumbu | Nechasalyan | 10306 | 42.92 | 37.76 | 5.17 | 7.55 |
| Solukhumbu | Solududhakunda | 10307 | 51.24 | 44.72 | 5.17 | 7.87 |
| Solukhumbu | Likhupike | 10308 | 50.08 | 43.52 | 5.08 | 7.52 |
| Okhaldhunga | Chisankhugadhi | 10401 | 48.00 | 41.66 | 5.17 | 7.40 |
| Okhaldhunga | Siddhicharan | 10402 | 54.55 | 47.91 | 5.17 | 8.41 |
| Okhaldhunga | Molung | 10403 | 52.55 | 46.31 | 5.17 | 8.41 |
| Okhaldhunga | Khijidemba | 10404 | 51.25 | 45.28 | 5.19 | 8.42 |
| Okhaldhunga | Likhu | 10405 | 51.64 | 45.58 | 5.19 | 8.42 |
| Okhaldhunga | Champadevi | 10406 | 52.52 | 46.29 | 5.16 | 8.40 |
| Okhaldhunga | Sunkoshi | 10407 | 54.93 | 48.22 | 5.17 | 8.41 |
| Okhaldhunga | Manebhanjyang | 10408 | 55.79 | 48.91 | 5.17 | 8.41 |
| Khotang | Kepilasagadhi | 10501 | 50.28 | 44.05 | 5.19 | 7.98 |
| Khotang | Ainselukhark | 10502 | 50.65 | 44.28 | 5.19 | 7.91 |
| Khotang | Rawa Besi | 10503 | 50.65 | 44.28 | 5.19 | 7.91 |
| Khotang | Halesi Tuwachung | 10504 | 51.39 | 45.42 | 5.19 | 8.46 |
| Khotang | Rupakot Majhuwagadhi | 10505 | 52.29 | 45.59 | 5.19 | 7.91 |
| Khotang | Sakela | 10506 | 50.41 | 44.09 | 5.19 | 7.91 |
| Khotang | Diprung | 10507 | 50.29 | 43.99 | 5.19 | 7.91 |
| Khotang | Khotehang | 10508 | 48.63 | 42.66 | 5.15 | 7.88 |
| Khotang | Jantedhunga | 10509 | 52.03 | 45.38 | 5.17 | 7.90 |
| Khotang | Barahapokhari | 10510 | 52.93 | 46.10 | 5.17 | 7.90 |
| Bhojpur | Shadananda | 10601 | 55.58 | 48.27 | 5.26 | 8.01 |
| Bhojpur | Salpasilichho | 10602 | 55.58 | 48.27 | 5.26 | 8.01 |
| Bhojpur | Tyamkemaiyung | 10603 | 50.79 | 44.44 | 5.19 | 7.96 |
| Bhojpur | Bhojpur | 10604 | 53.14 | 46.32 | 5.17 | 7.94 |
| Bhojpur | Arun | 10605 | 55.52 | 48.22 | 5.19 | 7.96 |
| Bhojpur | Pauwadungma | 10606 | 47.46 | 41.77 | 5.17 | 7.94 |
| Bhojpur | Ramprasad Rai | 10607 | 47.78 | 41.96 | 5.17 | 7.87 |
| Bhojpur | Hatuwagadhi | 10608 | 54.88 | 47.64 | 5.19 | 7.88 |
| Bhojpur | Aamchowk | 10609 | 54.65 | 47.45 | 5.17 | 7.87 |
| Dhankuta | Mahalaxmi | 10701 | 53.13 | 46.20 | 5.19 | 7.85 |
| Dhankuta | Pakhribas | 10702 | 52.71 | 45.86 | 5.33 | 7.96 |
| Dhankuta | Chhathar Jorpati | 10703 | 48.80 | 42.74 | 5.33 | 7.96 |
| Dhankuta | Dhankuta | 10704 | 52.99 | 46.02 | 5.33 | 7.89 |
| Dhankuta | Shahidbhumi | 10705 | 54.64 | 47.34 | 5.33 | 7.89 |
| Dhankuta | Sangurigadhi | 10706 | 53.06 | 46.07 | 5.72 | 8.20 |
| Dhankuta | Chaubise | 10707 | 55.76 | 48.08 | 5.18 | 7.61 |
| Terhathum | Aathrai | 10801 | 45.84 | 40.11 | 5.26 | 7.65 |
| Terhathum | Phedap | 10802 | 49.18 | 42.79 | 5.20 | 7.60 |
| Terhathum | Menchayam | 10803 | 50.17 | 43.81 | 5.20 | 7.83 |
| Terhathum | Myanglung | 10804 | 51.78 | 45.09 | 5.20 | 7.83 |
| Terhathum | Laligurans | 10805 | 51.87 | 45.17 | 5.20 | 7.83 |
| Terhathum | Chhathar | 10806 | 54.27 | 47.08 | 5.20 | 7.83 |
| Panchthar | Yangwarak | 10901 | 43.99 | 38.63 | 5.19 | 7.59 |
| Panchthar | Hilihang | 10902 | 50.33 | 43.70 | 5.26 | 7.65 |
| Panchthar | Falelung | 10903 | 49.32 | 42.90 | 5.26 | 7.65 |
| Panchthar | Phidim | 10904 | 51.59 | 44.71 | 5.28 | 7.67 |
| Panchthar | Falgunanda | 10905 | 51.59 | 44.71 | 5.28 | 7.67 |
| Panchthar | Kummayak | 10906 | 51.38 | 44.55 | 5.26 | 7.65 |
| Panchthar | Tumbewa | 10907 | 51.36 | 44.53 | 5.15 | 7.56 |
| Panchthar | Miklajung | 10908 | 56.79 | 48.88 | 5.23 | 7.62 |
| Ilam | Maijogmai | 11001 | 48.50 | 42.81 | 5.28 | 8.24 |
| Ilam | Sandakpur | 11002 | 51.21 | 44.99 | 5.28 | 8.24 |
| Ilam | Illam | 11003 | 52.91 | 46.35 | 5.28 | 8.24 |
| Ilam | Deumai | 11004 | 50.88 | 44.72 | 5.28 | 8.24 |
| Ilam | Fakphokthum | 11005 | 48.41 | 42.75 | 5.23 | 8.20 |
| Ilam | Mangsebung | 11006 | 58.70 | 50.98 | 5.20 | 8.18 |
| Ilam | Chulachuli | 11007 | 58.00 | 50.42 | 6.07 | 8.87 |
| Ilam | Mai | 11008 | 53.39 | 46.73 | 5.55 | 8.46 |
| Ilam | Suryodaya | 11009 | 49.67 | 43.76 | 5.55 | 8.46 |
| Ilam | Rong | 11010 | 55.54 | 47.77 | 5.58 | 7.81 |
| Jhapa | Mechinagar | 11101 | 61.41 | 54.93 | 9.05 | 13.05 |
| Jhapa | Buddhashanti | 11102 | 58.88 | 52.91 | 8.58 | 12.67 |
| Jhapa | Arjundhara | 11103 | 59.35 | 53.28 | 9.05 | 13.05 |
| Jhapa | Kankai | 11104 | 56.94 | 51.35 | 9.05 | 13.05 |
| Jhapa | Shivasataxi | 11105 | 58.89 | 53.59 | 8.51 | 13.28 |
| Jhapa | Kamal | 11106 | 61.35 | 55.56 | 9.07 | 13.73 |
| Jhapa | Damak | 11107 | 63.50 | 57.27 | 9.07 | 13.73 |
| Jhapa | Gauradhaha | 11108 | 61.59 | 55.07 | 8.74 | 12.79 |
| Jhapa | Gauriganj | 11109 | 58.83 | 52.87 | 8.45 | 12.56 |
| Jhapa | Jhapa | 11110 | 56.72 | 51.17 | 8.51 | 12.61 |
| Jhapa | Barhadashi | 11111 | 56.94 | 51.35 | 9.05 | 13.05 |
| Jhapa | Birtamod | 11112 | 59.35 | 53.28 | 9.05 | 13.05 |
| Jhapa | Haldibari | 11113 | 59.43 | 52.39 | 9.05 | 12.08 |
| Jhapa | Bhadrapur | 11114 | 60.35 | 54.08 | 10.05 | 13.85 |
| Jhapa | Kachankawal | 11115 | 59.42 | 52.38 | 8.69 | 11.79 |
| Morang | Miklajung | 11201 | 61.00 | 56.66 | 9.07 | 15.11 |
| Morang | Letang | 11202 | 58.42 | 54.59 | 8.79 | 14.89 |
| Morang | Kerabari | 11203 | 64.75 | 59.66 | 9.51 | 15.46 |
| Morang | Sundarharaicha | 11204 | 66.76 | 60.50 | 9.51 | 14.70 |
| Morang | Belbari | 11205 | 66.87 | 60.59 | 8.74 | 14.08 |
| Morang | Kanepokhari | 11206 | 65.23 | 59.28 | 8.79 | 14.12 |
| Morang | Patahrishanishchare | 11207 | 63.13 | 57.60 | 8.79 | 14.12 |
| Morang | Uralabari | 11208 | 61.00 | 56.66 | 9.07 | 15.11 |
| Morang | Ratuwamai | 11209 | 63.50 | 57.12 | 8.79 | 13.36 |
| Morang | Sunwarshi | 11210 | 65.32 | 58.58 | 8.79 | 13.36 |
| Morang | Rangeli | 11211 | 67.39 | 60.24 | 8.78 | 13.35 |
| Morang | Gramthan | 11212 | 68.87 | 61.52 | 11.17 | 15.36 |
| Morang | Budhiganga | 11213 | 69.22 | 61.80 | 11.17 | 15.36 |
| Morang | Biratnagar | 11214 | 73.13 | 64.93 | 13.17 | 16.96 |
| Morang | Katahari | 11215 | 68.87 | 61.42 | 11.17 | 15.26 |
| Morang | Dhanpalthan | 11216 | 67.99 | 60.72 | 8.78 | 13.35 |
| Morang | Jahada | 11217 | 68.87 | 61.42 | 11.17 | 15.26 |
| Sunsari | Dharan | 11301 | 67.94 | 58.02 | 8.51 | 10.47 |
| Sunsari | Barah | 11302 | 65.18 | 56.37 | 7.72 | 10.40 |
| Sunsari | Koshi | 11303 | 59.01 | 50.88 | 7.64 | 9.78 |
| Sunsari | Bhokraha Narsingh | 11304 | 64.66 | 55.39 | 7.82 | 9.93 |
| Sunsari | Ramdhuni | 11305 | 64.08 | 54.94 | 8.51 | 10.47 |
| Sunsari | Itahari | 11306 | 66.10 | 56.18 | 8.51 | 10.11 |
| Sunsari | Duhabi | 11307 | 68.54 | 57.47 | 10.17 | 10.77 |
| Sunsari | Gadhi | 11308 | 66.10 | 55.51 | 8.51 | 9.44 |
| Sunsari | Inaruwa | 11309 | 64.08 | 53.90 | 8.51 | 9.44 |
| Sunsari | Harinagar | 11310 | 67.29 | 56.47 | 7.82 | 8.89 |
| Sunsari | Dewanganj | 11311 | 68.45 | 57.39 | 7.79 | 8.87 |
| Sunsari | Barju | 11312 | 68.54 | 57.47 | 10.17 | 10.77 |
| Udayapur | Belaka | 11401 | 56.91 | 49.57 | 6.35 | 9.13 |
| Udayapur | Chaudandigadhi | 11402 | 53.31 | 46.69 | 6.35 | 9.13 |
| Udayapur | Triyuga | 11403 | 58.25 | 50.64 | 6.47 | 9.22 |
| Udayapur | Rautamai | 11404 | 54.27 | 47.46 | 6.16 | 8.97 |
| Udayapur | Sunkoshi | 11405 | 46.58 | 40.84 | 6.15 | 8.50 |
| Udayapur | Tapli | 11406 | 52.28 | 46.41 | 6.13 | 9.50 |
| Udayapur | Katari | 11407 | 58.93 | 51.74 | 6.55 | 9.83 |
| Udayapur | Udayapurgadhi | 11408 | 58.25 | 51.19 | 6.55 | 9.83 |
| Saptari | Saptakoshi | 20101 | 53.31 | 46.98 | 6.35 | 9.41 |
| Saptari | Kanchanrup | 20102 | 56.53 | 49.56 | 6.59 | 9.60 |
| Saptari | Agnisair Krishna Savaran | 20103 | 60.53 | 52.76 | 7.26 | 10.14 |
| Saptari | Rupani | 20104 | 60.53 | 52.20 | 7.26 | 9.58 |
| Saptari | Shambhunath | 20105 | 57.46 | 49.75 | 6.65 | 9.10 |
| Saptari | Khadak | 20106 | 56.40 | 48.90 | 6.74 | 9.16 |
| Saptari | Surunga | 20107 | 55.39 | 48.35 | 6.79 | 9.48 |
| Saptari | Balan Bihul | 20108 | 60.20 | 52.20 | 6.79 | 9.48 |
| Saptari | Bode Barsain | 20109 | 57.89 | 50.08 | 6.79 | 9.21 |
| Saptari | Dakneshwori | 20110 | 58.68 | 50.72 | 6.74 | 9.16 |
| Saptari | Belhi Chapena | 20111 | 59.46 | 51.34 | 6.74 | 9.16 |
| Saptari | Bishnupur | 20112 | 61.34 | 52.85 | 7.26 | 9.58 |
| Saptari | Rajbiraj | 20113 | 63.50 | 54.58 | 7.26 | 9.58 |
| Saptari | Mahadeva | 20114 | 61.34 | 52.85 | 7.26 | 9.58 |
| Saptari | Tirahut | 20115 | 59.50 | 51.38 | 6.82 | 9.23 |
| Saptari | Hanumannagar Kankalini | 20116 | 61.93 | 53.32 | 6.99 | 9.37 |
| Saptari | Tilathi Koiladi | 20117 | 61.34 | 52.57 | 7.26 | 9.30 |
| Saptari | Chhinnamasta | 20118 | 61.34 | 52.57 | 7.26 | 9.30 |
| Siraha | Lahan | 20201 | 59.79 | 52.01 | 6.64 | 9.49 |
| Siraha | Dhangadhimai | 20202 | 57.74 | 51.21 | 6.69 | 10.37 |
| Siraha | Golbazar | 20203 | 60.90 | 53.74 | 6.71 | 10.38 |
| Siraha | Mirchaiya | 20204 | 58.64 | 51.93 | 6.66 | 10.35 |
| Siraha | Karjanha | 20205 | 56.43 | 50.16 | 6.72 | 10.39 |
| Siraha | Kalyanpur | 20206 | 60.13 | 52.29 | 6.88 | 9.70 |
| Siraha | Naraha | 20207 | 59.23 | 51.58 | 6.71 | 9.56 |
| Siraha | Bishnupur | 20208 | 60.13 | 52.29 | 6.88 | 9.70 |
| Siraha | Arnama | 20209 | 60.13 | 52.29 | 6.88 | 9.70 |
| Siraha | Sukhipur | 20210 | 59.12 | 51.47 | 6.69 | 9.53 |
| Siraha | Laxmipur Patari | 20211 | 58.09 | 50.65 | 6.69 | 9.53 |
| Siraha | Sakhuwanankarkatti | 20212 | 57.84 | 50.45 | 6.79 | 9.61 |
| Siraha | Bhagawanpur | 20213 | 57.77 | 50.40 | 6.79 | 9.61 |
| Siraha | Nawarajpur | 20214 | 60.39 | 52.49 | 6.67 | 9.52 |
| Siraha | Bariyarpatti | 20215 | 61.11 | 53.07 | 6.69 | 9.53 |
| Siraha | Aurahi | 20216 | 60.13 | 52.29 | 6.88 | 9.70 |
| Siraha | Siraha | 20217 | 62.28 | 54.02 | 6.91 | 9.72 |
| Dhanusha | Ganeshman Charnath | 20301 | 56.31 | 49.91 | 8.72 | 11.83 |
| Dhanusha | Dhanusadham | 20302 | 58.91 | 51.99 | 8.90 | 11.98 |
| Dhanusha | Mithila | 20303 | 55.60 | 49.34 | 8.84 | 11.93 |
| Dhanusha | Bateshwor | 20304 | 63.07 | 55.31 | 8.93 | 12.00 |
| Dhanusha | Chhireshwornath | 20305 | 66.10 | 57.74 | 8.93 | 12.00 |
| Dhanusha | Lakshminiya | 20306 | 64.26 | 55.53 | 9.90 | 12.04 |
| Dhanusha | Mithila Bihari | 20307 | 61.63 | 53.43 | 9.90 | 12.04 |
| Dhanusha | Hansapur | 20308 | 64.26 | 55.53 | 9.90 | 12.04 |
| Dhanusha | Sabaila | 20309 | 58.15 | 51.38 | 8.81 | 11.91 |
| Dhanusha | Sahidnagar | 20310 | 60.47 | 52.41 | 8.89 | 11.14 |
| Dhanusha | Kamala | 20311 | 61.75 | 53.43 | 8.91 | 11.16 |
| Dhanusha | Janaknandani | 20312 | 61.75 | 53.43 | 8.91 | 11.16 |
| Dhanusha | Bideha | 20313 | 61.18 | 52.98 | 8.97 | 11.21 |
| Dhanusha | Aaurahi | 20314 | 64.26 | 55.53 | 9.90 | 12.04 |
| Dhanusha | Janakpur | 20315 | 67.24 | 57.91 | 10.90 | 12.84 |
| Dhanusha | Dhanauji | 20316 | 64.26 | 55.53 | 9.90 | 12.04 |
| Dhanusha | Nagarain | 20317 | 64.26 | 55.53 | 9.90 | 12.04 |
| Dhanusha | Mukhiyapatti Musarmiya | 20318 | 63.59 | 54.99 | 9.07 | 11.38 |
| Mahottari | Bardibas | 20401 | 59.22 | 52.38 | 5.89 | 9.72 |
| Mahottari | Gaushala | 20402 | 57.98 | 51.38 | 6.19 | 9.95 |
| Mahottari | Sonama | 20403 | 58.36 | 50.97 | 6.57 | 9.54 |
| Mahottari | Aurahi | 20404 | 60.53 | 53.43 | 6.57 | 10.26 |
| Mahottari | Bhangaha | 20405 | 61.74 | 54.40 | 5.96 | 9.77 |
| Mahottari | Loharpatti | 20406 | 60.78 | 52.88 | 6.90 | 9.78 |
| Mahottari | Balwa | 20407 | 60.56 | 52.71 | 6.00 | 9.07 |
| Mahottari | Ramgopalpur | 20408 | 61.13 | 53.17 | 6.57 | 9.52 |
| Mahottari | Samsi | 20409 | 58.89 | 51.39 | 6.57 | 9.54 |
| Mahottari | Manra Siswa | 20410 | 58.59 | 51.14 | 6.57 | 9.52 |
| Mahottari | Ekdanra | 20411 | 58.35 | 50.94 | 6.33 | 9.33 |
| Mahottari | Mahottari | 20412 | 60.55 | 52.70 | 6.33 | 9.33 |
| Mahottari | Pipra | 20413 | 60.78 | 52.88 | 6.90 | 9.78 |
| Mahottari | Matihani | 20414 | 62.38 | 54.17 | 6.33 | 9.33 |
| Mahottari | Jaleswor | 20415 | 60.55 | 52.70 | 6.33 | 9.33 |
| Sarlahi | Lalbandi | 20501 | 58.10 | 50.72 | 6.56 | 9.49 |
| Sarlahi | Hariwan | 20502 | 60.46 | 52.61 | 6.56 | 9.49 |
| Sarlahi | Bagmati | 20503 | 57.31 | 50.09 | 6.65 | 9.57 |
| Sarlahi | Barahathawa | 20504 | 58.58 | 51.11 | 7.12 | 9.93 |
| Sarlahi | Haripur | 20505 | 58.30 | 50.88 | 6.88 | 9.75 |
| Sarlahi | Ishworpur | 20506 | 56.11 | 49.13 | 7.19 | 9.99 |
| Sarlahi | Haripurwa | 20507 | 53.73 | 47.23 | 7.19 | 9.99 |
| Sarlahi | Parsa | 20508 | 56.73 | 48.90 | 7.19 | 9.27 |
| Sarlahi | Bramhapuri | 20509 | 56.77 | 49.66 | 7.53 | 10.27 |
| Sarlahi | Chandranagar | 20510 | 56.12 | 49.14 | 6.88 | 9.75 |
| Sarlahi | Kabilasi | 20511 | 56.55 | 48.75 | 7.53 | 9.54 |
| Sarlahi | Chakraghatta | 20512 | 60.87 | 52.21 | 7.53 | 9.54 |
| Sarlahi | Basbariya | 20513 | 58.81 | 50.57 | 7.12 | 9.21 |
| Sarlahi | Dhankaul | 20514 | 54.62 | 47.21 | 7.09 | 9.19 |
| Sarlahi | Ramnagar | 20515 | 60.58 | 51.98 | 7.15 | 9.23 |
| Sarlahi | Balara | 20516 | 62.83 | 53.78 | 7.15 | 9.23 |
| Sarlahi | Godaita | 20517 | 57.38 | 49.42 | 7.12 | 9.21 |
| Sarlahi | Bishnu | 20518 | 59.92 | 51.46 | 7.03 | 9.14 |
| Sarlahi | Kaudena | 20519 | 59.37 | 51.02 | 7.53 | 9.54 |
| Sarlahi | Malangawa | 20520 | 59.42 | 51.05 | 7.53 | 9.54 |
| Rautahat | Chandrapur | 20601 | 59.62 | 52.53 | 6.45 | 9.99 |
| Rautahat | Gujara | 20602 | 63.25 | 55.43 | 6.95 | 10.39 |
| Rautahat | Phatuwa Bijayapur | 20603 | 64.26 | 55.39 | 6.95 | 9.54 |
| Rautahat | Katahariya | 20604 | 58.77 | 51.12 | 7.14 | 9.82 |
| Rautahat | Brindaban | 20605 | 58.16 | 51.35 | 7.14 | 10.54 |
| Rautahat | Gadhimai | 20606 | 56.70 | 49.46 | 7.14 | 9.82 |
| Rautahat | Madhav Narayan | 20607 | 58.90 | 51.22 | 7.44 | 10.05 |
| Rautahat | Garuda | 20608 | 58.77 | 51.12 | 7.14 | 9.82 |
| Rautahat | Dewahhi Gonahi | 20609 | 58.60 | 50.99 | 7.14 | 9.82 |
| Rautahat | Maulapur | 20610 | 56.60 | 49.26 | 7.21 | 9.75 |
| Rautahat | Baudhimai | 20611 | 57.64 | 50.10 | 7.21 | 9.75 |
| Rautahat | Paroha | 20612 | 58.19 | 50.65 | 7.44 | 10.05 |
| Rautahat | Rajpur | 20613 | 59.45 | 51.66 | 7.63 | 10.21 |
| Rautahat | Yemunamai | 20614 | 61.56 | 53.35 | 7.44 | 10.05 |
| Rautahat | Durga Bhagwati | 20615 | 61.36 | 53.19 | 7.44 | 10.05 |
| Rautahat | Rajdevi | 20616 | 61.55 | 53.34 | 7.63 | 10.21 |
| Rautahat | Gaur | 20617 | 59.45 | 51.66 | 7.63 | 10.21 |
| Rautahat | Ishanath | 20618 | 63.16 | 54.51 | 7.63 | 10.08 |
| Bara | Nijgadh | 20701 | 64.02 | 54.93 | 7.59 | 9.78 |
| Bara | Kolhabi | 20702 | 62.29 | 53.55 | 7.69 | 9.87 |
| Bara | Jitpur Simara | 20703 | 64.65 | 55.43 | 8.83 | 10.78 |
| Bara | Parwanipur | 20704 | 64.65 | 55.08 | 8.83 | 10.42 |
| Bara | Prasauni | 20705 | 64.65 | 55.08 | 8.83 | 10.42 |
| Bara | Bishrampur | 20706 | 64.65 | 55.00 | 8.83 | 10.34 |
| Bara | Pheta | 20707 | 65.39 | 55.59 | 8.21 | 9.85 |
| Bara | Kalaiya | 20708 | 63.12 | 53.86 | 8.21 | 9.93 |
| Bara | Karaiyamai | 20709 | 63.04 | 54.14 | 8.13 | 10.22 |
| Bara | Baragadhi | 20710 | 62.33 | 53.58 | 7.78 | 9.94 |
| Bara | Adarshkotwal | 20711 | 62.45 | 53.67 | 8.21 | 10.28 |
| Bara | Simraungadh | 20712 | 60.21 | 51.88 | 8.21 | 10.28 |
| Bara | Pacharauta | 20713 | 62.64 | 53.83 | 8.21 | 10.28 |
| Bara | Mahagadhimai | 20714 | 61.00 | 52.08 | 8.13 | 9.78 |
| Bara | Devtal | 20715 | 61.00 | 52.08 | 8.13 | 9.78 |
| Bara | Suwarna | 20716 | 62.43 | 53.22 | 8.00 | 9.68 |
| Parsa | Thori | 20801 | 55.60 | 48.61 | 8.41 | 10.86 |
| Parsa | Jirabhawani | 20802 | 56.55 | 49.36 | 8.70 | 11.09 |
| Parsa | Jagarnathpur | 20803 | 67.55 | 57.89 | 9.02 | 11.06 |
| Parsa | Paterwasugauli | 20804 | 67.27 | 57.94 | 8.70 | 11.09 |
| Parsa | SakhuwaPrasauni | 20805 | 67.31 | 57.97 | 9.26 | 11.54 |
| Parsa | Parsagadhi | 20806 | 65.97 | 56.90 | 9.83 | 11.99 |
| Parsa | Birgunj | 20807 | 69.88 | 60.03 | 11.83 | 13.59 |
| Parsa | Bahudaramai | 20808 | 65.97 | 56.82 | 9.83 | 11.91 |
| Parsa | Pokhariya | 20809 | 69.60 | 59.53 | 9.26 | 11.25 |
| Parsa | Kalikamai | 20810 | 67.61 | 57.93 | 9.07 | 11.11 |
| Parsa | Dhobini | 20811 | 67.72 | 58.02 | 9.07 | 11.11 |
| Parsa | Chhipaharmai | 20812 | 57.44 | 49.80 | 9.07 | 11.11 |
| Parsa | Pakahamainpur | 20813 | 67.22 | 57.62 | 9.07 | 11.11 |
| Parsa | Bindabasini | 20814 | 65.97 | 56.82 | 9.83 | 11.91 |
| Dolakha | Gaurishankar | 30101 | 44.55 | 40.05 | 5.25 | 8.61 |
| Dolakha | Bigu | 30102 | 51.00 | 44.96 | 5.20 | 8.32 |
| Dolakha | Kalinchok | 30103 | 52.15 | 45.87 | 5.25 | 8.35 |
| Dolakha | Baiteshwor | 30104 | 44.77 | 39.97 | 5.25 | 8.35 |
| Dolakha | Jiri | 30105 | 45.28 | 40.37 | 5.25 | 8.34 |
| Dolakha | Tamakoshi | 30106 | 46.65 | 42.21 | 5.25 | 9.09 |
| Dolakha | Melung | 30107 | 44.38 | 40.39 | 5.23 | 9.08 |
| Dolakha | Sailung | 30108 | 44.77 | 39.97 | 5.25 | 8.35 |
| Dolakha | Bhimeshwor | 30109 | 52.15 | 45.87 | 5.25 | 8.35 |
| Sindhupalchok | Bhotekoshi | 30201 | 50.80 | 44.93 | 5.20 | 8.46 |
| Sindhupalchok | Jugal | 30202 | 52.40 | 46.21 | 5.31 | 8.55 |
| Sindhupalchok | Panchpokhari Thangpal | 30203 | 47.25 | 42.10 | 5.28 | 8.52 |
| Sindhupalchok | Helambu | 30204 | 67.95 | 58.65 | 5.28 | 8.52 |
| Sindhupalchok | Melamchi | 30205 | 67.14 | 58.01 | 6.84 | 9.77 |
| Sindhupalchok | Indrawati | 30206 | 64.21 | 55.66 | 5.37 | 8.59 |
| Sindhupalchok | Chautara SangachokGadhi | 30207 | 48.61 | 43.19 | 5.37 | 8.59 |
| Sindhupalchok | Balefi | 30208 | 51.96 | 45.86 | 5.31 | 8.55 |
| Sindhupalchok | Barhabise | 30209 | 52.83 | 46.56 | 5.31 | 8.54 |
| Sindhupalchok | Tripurasundari | 30210 | 54.95 | 48.26 | 5.31 | 8.54 |
| Sindhupalchok | Lisangkhu Pakhar | 30211 | 52.14 | 46.01 | 5.24 | 8.49 |
| Sindhupalchok | Sunkoshi | 30212 | 51.96 | 45.86 | 5.31 | 8.55 |
| Rasuwa | Gosaikunda | 30301 | 62.62 | 53.38 | 5.22 | 7.46 |
| Rasuwa | Parbati Kunda | 30302 | 53.94 | 46.97 | 5.07 | 7.87 |
| Rasuwa | Uttargaya | 30303 | 61.33 | 52.88 | 5.42 | 8.15 |
| Rasuwa | Kalika | 30304 | 62.37 | 53.18 | 5.42 | 7.62 |
| Rasuwa | Naukunda | 30305 | 65.24 | 55.48 | 5.34 | 7.56 |
| Dhading | Rubi Valley | 30401 | 59.21 | 52.23 | 6.15 | 9.79 |
| Dhading | Khaniyabash | 30402 | 62.30 | 54.71 | 6.18 | 9.81 |
| Dhading | Gangajamuna | 30403 | 59.74 | 52.65 | 6.27 | 9.88 |
| Dhading | Tripura Sundari | 30404 | 55.81 | 49.51 | 6.29 | 9.90 |
| Dhading | Netrawati Dabjong | 30405 | 58.01 | 51.27 | 6.29 | 9.90 |
| Dhading | Nilakantha | 30406 | 53.61 | 47.75 | 6.29 | 9.90 |
| Dhading | Jwalamukhi | 30407 | 61.97 | 54.44 | 6.29 | 9.90 |
| Dhading | Siddhalek | 30408 | 61.97 | 54.44 | 6.29 | 9.90 |
| Dhading | Benighat Rorang | 30409 | 63.96 | 56.03 | 6.28 | 9.89 |
| Dhading | Gajuri | 30410 | 67.44 | 58.81 | 6.23 | 9.85 |
| Dhading | Galchi | 30411 | 66.60 | 57.18 | 6.34 | 8.97 |
| Dhading | Thakre | 30412 | 69.83 | 59.76 | 6.34 | 8.97 |
| Dhading | Dhunibesi | 30413 | 71.86 | 61.39 | 8.34 | 10.57 |
| Nuwakot | Dupcheshwar | 30501 | 67.59 | 58.09 | 5.30 | 8.27 |
| Nuwakot | Tadi | 30502 | 67.08 | 57.69 | 5.34 | 8.30 |
| Nuwakot | Suryagadhi | 30503 | 64.22 | 55.36 | 5.42 | 8.31 |
| Nuwakot | Bidur | 30504 | 64.22 | 55.36 | 5.42 | 8.31 |
| Nuwakot | Kispang | 30505 | 61.33 | 53.04 | 5.42 | 8.31 |
| Nuwakot | Meghang | 30506 | 56.76 | 49.39 | 5.42 | 8.31 |
| Nuwakot | Tarkeshwar | 30507 | 56.25 | 48.98 | 5.42 | 8.31 |
| Nuwakot | Belkotgadhi | 30508 | 67.98 | 58.36 | 5.42 | 8.31 |
| Nuwakot | Likhu | 30509 | 66.81 | 57.47 | 5.42 | 8.36 |
| Nuwakot | Panchakanya | 30510 | 69.54 | 59.66 | 5.35 | 8.31 |
| Nuwakot | Shivapuri | 30511 | 69.90 | 59.95 | 5.35 | 8.31 |
| Nuwakot | Kakani | 30512 | 70.89 | 60.74 | 7.34 | 9.90 |
| Kathmandu | Shankharapur | 30601 | 72.06 | 72.84 | 12.21 | 24.96 |
| Kathmandu | Kageshwori Manahora | 30602 | 89.68 | 86.93 | 30.00 | 39.19 |
| Kathmandu | Gokarneshwor | 30603 | 88.68 | 86.13 | 29.00 | 38.39 |
| Kathmandu | Budhanilakantha | 30604 | 88.55 | 86.03 | 30.00 | 39.19 |
| Kathmandu | Tokha | 30605 | 88.55 | 86.03 | 30.00 | 39.19 |
| Kathmandu | Tarakeshwor | 30606 | 89.44 | 86.75 | 30.00 | 39.19 |
| Kathmandu | Nagarjun | 30607 | 89.44 | 86.31 | 30.00 | 38.76 |
| Kathmandu | Kathmandu | 30608 | 91.37 | 88.29 | 30.00 | 39.19 |
| Kathmandu | Kirtipur | 30609 | 88.96 | 85.93 | 30.00 | 38.76 |
| Kathmandu | Chandragiri | 30610 | 80.10 | 78.84 | 14.46 | 26.33 |
| Kathmandu | Dakshinkali | 30611 | 75.69 | 75.31 | 15.92 | 27.49 |
| Bhaktapur | Changunarayan | 30701 | 79.24 | 68.40 | 20.51 | 21.42 |
| Bhaktapur | Bhaktapur | 30702 | 82.79 | 70.64 | 22.51 | 22.42 |
| Bhaktapur | Madhyapur Thimi | 30703 | 89.96 | 76.38 | 30.00 | 28.41 |
| Bhaktapur | Suryabinayak | 30704 | 88.77 | 75.43 | 22.51 | 22.42 |
| Lalitpur | Mahalaxmi | 30801 | 89.68 | 79.08 | 30.00 | 31.33 |
| Lalitpur | Lalitpur | 30802 | 89.68 | 79.08 | 30.00 | 31.33 |
| Lalitpur | Godawari | 30803 | 72.72 | 65.51 | 15.92 | 20.07 |
| Lalitpur | Konjyosom | 30804 | 53.35 | 50.01 | 5.82 | 11.99 |
| Lalitpur | Mahankal | 30805 | 58.78 | 55.56 | 5.40 | 12.85 |
| Lalitpur | Bagmati | 30806 | 65.32 | 60.79 | 5.82 | 13.19 |
| Kavre | Chaurideurali | 30901 | 46.29 | 44.43 | 8.26 | 14.00 |
| Kavre | Bhumlu | 30902 | 51.61 | 48.69 | 8.37 | 14.09 |
| Kavre | Mandandeupur | 30903 | 68.40 | 61.38 | 9.02 | 13.88 |
| Kavre | Banepa | 30904 | 65.65 | 59.18 | 9.86 | 14.54 |
| Kavre | Dhulikhel | 30905 | 9.02 | 13.21 | 9.02 | 13.21 |
| Kavre | Panchkhal | 30906 | 9.02 | 14.61 | 9.02 | 14.61 |
| Kavre | Temal | 30907 | 55.69 | 51.94 | 8.37 | 14.09 |
| Kavre | Namobuddha | 30908 | 8.59 | 14.27 | 8.59 | 14.27 |
| Kavre | Panauti | 30909 | 66.15 | 59.11 | 10.35 | 14.48 |
| Kavre | Bethanchowk | 30910 | 52.41 | 49.32 | 8.40 | 14.11 |
| Kavre | Roshi | 30911 | 53.76 | 50.41 | 8.29 | 14.03 |
| Kavre | Mahabharat | 30912 | 58.03 | 53.82 | 8.16 | 13.93 |
| Kavre | Khanikhola | 30913 | 55.35 | 51.67 | 8.17 | 13.93 |
| Ramechhap | Umakunda | 31001 | 54.96 | 48.10 | 6.19 | 9.08 |
| Ramechhap | Gokulganga | 31002 | 46.10 | 40.73 | 6.23 | 8.84 |
| Ramechhap | Likhu Tamakoshi | 31003 | 52.91 | 46.93 | 6.23 | 9.59 |
| Ramechhap | Ramechhap | 31004 | 54.56 | 48.25 | 6.22 | 9.58 |
| Ramechhap | Manthali | 31005 | 52.53 | 46.63 | 6.23 | 9.59 |
| Ramechhap | Khadadevi | 31006 | 53.71 | 47.57 | 6.26 | 9.61 |
| Ramechhap | Doramba | 31007 | 46.53 | 41.83 | 6.23 | 9.59 |
| Ramechhap | Sunapati | 31008 | 56.54 | 49.83 | 6.26 | 9.61 |
| Sindhuli | Dudhouli | 31101 | 52.19 | 46.25 | 6.17 | 9.43 |
| Sindhuli | Phikkal | 31102 | 54.37 | 47.99 | 6.17 | 9.43 |
| Sindhuli | Tinpatan | 31103 | 51.83 | 45.96 | 6.17 | 9.43 |
| Sindhuli | Golanjor | 31104 | 51.88 | 46.00 | 6.22 | 9.47 |
| Sindhuli | Kamalamai | 31105 | 55.39 | 48.81 | 6.37 | 9.59 |
| Sindhuli | Sunkoshi | 31106 | 53.24 | 47.09 | 6.26 | 9.50 |
| Sindhuli | Ghanglekh | 31107 | 55.12 | 48.59 | 6.16 | 9.43 |
| Sindhuli | Marin | 31108 | 57.39 | 50.40 | 6.25 | 9.50 |
| Sindhuli | Hariharpurgadhi | 31109 | 57.12 | 50.19 | 6.46 | 9.67 |
| Makawanpur | Indrasarowar | 31201 | 71.47 | 60.46 | 7.95 | 9.64 |
| Makawanpur | Thaha | 31202 | 69.71 | 59.05 | 7.95 | 9.64 |
| Makawanpur | Kailash | 31203 | 64.85 | 56.13 | 6.59 | 9.52 |
| Makawanpur | Raksirang | 31204 | 63.14 | 54.76 | 6.59 | 9.52 |
| Makawanpur | Manahari | 31205 | 63.14 | 53.79 | 6.59 | 8.55 |
| Makawanpur | Hetauda | 31206 | 65.13 | 55.71 | 6.59 | 8.87 |
| Makawanpur | Bhimphedi | 31207 | 64.85 | 55.48 | 6.59 | 8.87 |
| Makawanpur | Makawanpurgadhi | 31208 | 63.14 | 54.11 | 6.59 | 8.87 |
| Makawanpur | Bakaiya | 31209 | 63.02 | 54.87 | 6.59 | 9.72 |
| Makawanpur | Bagmati | 31210 | 56.95 | 50.01 | 6.29 | 9.48 |
| Chitawan | Rapti | 31301 | 66.34 | 63.67 | 8.66 | 17.52 |
| Chitawan | Kalika | 31302 | 65.22 | 62.77 | 9.02 | 17.81 |
| Chitawan | Ichchhyakamana | 31303 | 64.85 | 62.47 | 8.65 | 17.52 |
| Chitawan | Bharatpur | 31304 | 66.36 | 63.68 | 9.02 | 17.81 |
| Chitawan | Ratnanagar | 31305 | 58.22 | 56.38 | 9.02 | 17.02 |
| Chitawan | Khairahani | 31306 | 58.83 | 56.70 | 9.02 | 16.85 |
| Chitawan | Madi | 31307 | 57.29 | 55.43 | 8.17 | 16.14 |
| Gorkha | Chum Nubri | 40101 | 54.14 | 48.67 | 6.13 | 10.26 |
| Gorkha | Ajirkot | 40102 | 57.02 | 50.98 | 6.19 | 10.32 |
| Gorkha | Sulikot | 40103 | 53.00 | 47.76 | 6.22 | 10.34 |
| Gorkha | Dharche | 40104 | 57.26 | 51.17 | 6.15 | 10.28 |
| Gorkha | Aarughat | 40105 | 58.19 | 51.92 | 6.27 | 10.38 |
| Gorkha | Bhimsen | 40106 | 62.06 | 55.01 | 6.37 | 10.46 |
| Gorkha | Siranchok | 40107 | 59.19 | 52.71 | 6.37 | 10.46 |
| Gorkha | Palungtar | 40108 | 64.16 | 56.69 | 6.37 | 10.46 |
| Gorkha | Gorkha | 40109 | 62.90 | 55.68 | 6.37 | 10.46 |
| Gorkha | Sahid Lakhan | 40110 | 64.30 | 56.80 | 6.37 | 10.46 |
| Gorkha | Gandaki | 40111 | 65.31 | 57.61 | 6.22 | 10.34 |
| Manang | Narphu | 40201 | 58.01 | 50.70 | 5.01 | 8.30 |
| Manang | Neshyang | 40202 | 43.61 | 39.18 | 5.06 | 8.33 |
| Manang | Chame | 40203 | 53.57 | 46.58 | 5.03 | 7.75 |
| Manang | Nashong | 40204 | 56.43 | 48.87 | 5.03 | 7.75 |
| Mustang | Dalome | 40301 | 5.00 | 8.29 | 5.00 | 8.29 |
| Mustang | Gharapjhong | 40302 | 43.83 | 39.36 | 5.02 | 8.31 |
| Mustang | Barhagaun Muktikhsetra | 40303 | 32.43 | 30.23 | 5.02 | 8.30 |
| Mustang | Lomanthang | 40304 | 5.00 | 8.29 | 5.00 | 8.29 |
| Mustang | Thasang | 40305 | 48.28 | 42.92 | 5.04 | 8.32 |
| Myagdi | Annapurna | 40401 | 63.78 | 53.76 | 5.26 | 6.94 |
| Myagdi | Raghuganga | 40402 | 50.71 | 45.29 | 5.44 | 9.07 |
| Myagdi | Dhaulagiri | 40403 | 51.94 | 46.27 | 5.13 | 8.83 |
| Myagdi | Malika | 40404 | 55.83 | 49.38 | 5.18 | 8.87 |
| Myagdi | Mangala | 40405 | 57.99 | 49.12 | 5.44 | 7.08 |
| Myagdi | Beni | 40406 | 59.33 | 50.19 | 5.59 | 7.20 |
| Kaski | Madi | 40501 | 61.43 | 61.60 | 7.92 | 18.79 |
| Kaski | Machhapuchchhre | 40502 | 62.57 | 61.72 | 8.92 | 18.80 |
| Kaski | Annapurna | 40503 | 62.99 | 62.05 | 8.92 | 18.80 |
| Kaski | Pokhara Lekhnath | 40504 | 64.50 | 63.27 | 8.92 | 18.80 |
| Kaski | Rupa | 40505 | 61.57 | 60.92 | 7.92 | 18.00 |
| Lamjung | Dordi | 40601 | 58.11 | 51.12 | 5.37 | 8.93 |
| Lamjung | Marsyangdi | 40602 | 58.05 | 51.07 | 5.31 | 8.88 |
| Lamjung | Kwholasothar | 40603 | 58.05 | 51.07 | 5.31 | 8.88 |
| Lamjung | MadhyaNepal | 40604 | 58.36 | 50.53 | 5.37 | 8.14 |
| Lamjung | Besishahar | 40605 | 60.41 | 52.96 | 5.37 | 8.93 |
| Lamjung | Sundarbazar | 40606 | 58.36 | 51.32 | 5.37 | 8.93 |
| Lamjung | Rainas | 40607 | 59.20 | 51.99 | 5.37 | 8.93 |
| Lamjung | Dudhpokhari | 40608 | 59.05 | 51.88 | 5.26 | 8.84 |
| Tanahu | Bhanu | 40701 | 61.16 | 53.35 | 6.37 | 9.52 |
| Tanahu | Byas | 40702 | 63.32 | 55.08 | 6.37 | 9.52 |
| Tanahu | Myagde | 40703 | 60.72 | 52.21 | 6.29 | 8.67 |
| Tanahu | Shuklagandaki | 40704 | 60.87 | 52.33 | 6.92 | 9.17 |
| Tanahu | Bhimad | 40705 | 62.06 | 53.28 | 6.29 | 8.67 |
| Tanahu | Ghiring | 40706 | 62.70 | 53.79 | 6.29 | 8.66 |
| Tanahu | Rhishing | 40707 | 62.97 | 54.01 | 6.28 | 8.66 |
| Tanahu | Devghat | 40708 | 62.41 | 54.36 | 6.65 | 9.74 |
| Tanahu | Bandipur | 40709 | 62.87 | 54.72 | 6.28 | 9.45 |
| Tanahu | Anbukhaireni | 40710 | 62.10 | 54.10 | 6.65 | 9.74 |
| Nawalparasi_E | Gaidakot | 40801 | 61.08 | 52.32 | 5.65 | 7.97 |
| Nawalparasi_E | Bulingtar | 40802 | 62.48 | 53.44 | 5.38 | 7.76 |
| Nawalparasi_E | Bungdikali | 40803 | 62.45 | 53.41 | 5.17 | 7.59 |
| Nawalparasi_E | Hupsekot | 40804 | 60.03 | 51.48 | 5.58 | 7.92 |
| Nawalparasi_E | Devchuli | 40805 | 62.17 | 53.19 | 5.58 | 7.92 |
| Nawalparasi_E | Kawasoti | 40806 | 60.03 | 51.00 | 5.58 | 7.43 |
| Nawalparasi_E | Madhyabindu | 40807 | 58.21 | 50.02 | 5.58 | 7.92 |
| Nawalparasi_E | Binayee Tribeni | 40808 | 58.54 | 50.29 | 5.32 | 7.71 |
| Syangja | Putalibazar | 40901 | 61.18 | 53.00 | 6.92 | 9.59 |
| Syangja | Phedikhola | 40902 | 61.18 | 53.00 | 6.92 | 9.59 |
| Syangja | Aandhikhola | 40903 | 61.18 | 53.00 | 6.92 | 9.59 |
| Syangja | Arjunchaupari | 40904 | 60.06 | 52.10 | 6.36 | 9.15 |
| Syangja | Bhirkot | 40905 | 55.72 | 48.63 | 6.40 | 9.18 |
| Syangja | Biruwa | 40906 | 57.95 | 50.42 | 6.40 | 9.18 |
| Syangja | Harinas | 40907 | 55.32 | 48.31 | 6.29 | 9.09 |
| Syangja | Chapakot | 40908 | 56.39 | 49.17 | 6.40 | 9.18 |
| Syangja | Waling | 40909 | 55.72 | 48.63 | 6.40 | 9.18 |
| Syangja | Galyang | 40910 | 56.39 | 49.17 | 6.40 | 9.18 |
| Syangja | Kaligandagi | 40911 | 57.10 | 49.25 | 6.46 | 8.74 |
| Parbat | Modi | 41001 | 59.99 | 51.55 | 5.92 | 8.29 |
| Parbat | Jaljala | 41002 | 61.53 | 52.14 | 5.59 | 7.39 |
| Parbat | Kushma | 41003 | 60.02 | 51.57 | 5.92 | 8.29 |
| Parbat | Phalebas | 41004 | 61.53 | 52.14 | 5.59 | 7.39 |
| Parbat | Mahashila | 41005 | 58.54 | 49.75 | 5.36 | 7.21 |
| Parbat | Bihadi | 41006 | 60.03 | 51.10 | 5.36 | 7.36 |
| Parbat | Painyu | 41007 | 54.55 | 46.71 | 5.40 | 7.39 |
| Baglung | Baglung | 41101 | 61.90 | 52.74 | 5.59 | 7.69 |
| Baglung | Kanthekhola | 41102 | 59.69 | 50.98 | 5.59 | 7.69 |
| Baglung | Tara Khola | 41103 | 58.24 | 49.79 | 5.28 | 7.42 |
| Baglung | Taman Khola | 41104 | 54.34 | 48.68 | 5.13 | 9.32 |
| Baglung | Dhorpatan | 41105 | 55.92 | 49.22 | 5.17 | 8.62 |
| Baglung | Nisikhola | 41106 | 57.45 | 50.44 | 5.18 | 8.63 |
| Baglung | Badigad | 41107 | 54.45 | 48.04 | 5.29 | 8.71 |
| Baglung | Galkot | 41108 | 58.25 | 49.82 | 5.29 | 7.45 |
| Baglung | Bareng | 41109 | 58.23 | 49.81 | 5.27 | 7.43 |
| Baglung | Jaimuni | 41110 | 59.69 | 50.98 | 5.59 | 7.69 |
| Rukum_E | Putha Uttarganga | 50101 | 53.07 | 46.81 | 5.07 | 8.41 |
| Rukum_E | Sisne | 50102 | 55.67 | 49.25 | 5.24 | 8.91 |
| Rukum_E | Bhume | 50103 | 51.02 | 45.17 | 5.24 | 8.55 |
| Rolpa | Sunchhahari | 50201 | 55.59 | 48.34 | 5.18 | 8.01 |
| Rolpa | Thawang | 50202 | 53.19 | 46.42 | 5.13 | 7.97 |
| Rolpa | Duikholi | 50203 | 51.80 | 46.06 | 5.24 | 8.81 |
| Rolpa | Sukidaha | 50204 | 54.82 | 48.47 | 5.23 | 8.80 |
| Rolpa | Madi | 50205 | 53.23 | 47.20 | 5.16 | 8.75 |
| Rolpa | Tribeni | 50206 | 53.23 | 47.20 | 5.16 | 8.75 |
| Rolpa | Rolpa | 50207 | 55.59 | 49.09 | 5.18 | 8.76 |
| Rolpa | Runtigadi | 50208 | 58.41 | 51.34 | 5.37 | 8.91 |
| Rolpa | Suwarnabati | 50209 | 57.74 | 50.81 | 5.30 | 8.86 |
| Rolpa | Lungri | 50210 | 57.74 | 50.05 | 5.30 | 8.10 |
| Pyuthan | Gaumukhi | 50301 | 57.38 | 50.27 | 6.26 | 9.37 |
| Pyuthan | Naubahini | 50302 | 58.35 | 51.05 | 6.30 | 9.40 |
| Pyuthan | Jhimruk | 50303 | 57.62 | 50.29 | 6.30 | 9.23 |
| Pyuthan | Pyuthan | 50304 | 60.66 | 53.43 | 6.30 | 9.94 |
| Pyuthan | Sworgadwary | 50305 | 61.57 | 54.37 | 6.30 | 10.16 |
| Pyuthan | Mandavi | 50306 | 59.89 | 52.81 | 6.30 | 9.94 |
| Pyuthan | Mallarani | 50307 | 59.93 | 52.84 | 6.30 | 9.94 |
| Pyuthan | Ayirabati | 50308 | 61.88 | 54.40 | 6.22 | 9.88 |
| Pyuthan | Sarumarani | 50309 | 61.87 | 54.40 | 6.25 | 9.90 |
| Gulmi | Kaligandaki | 50401 | 58.66 | 49.98 | 6.30 | 8.10 |
| Gulmi | Satyawati | 50402 | 56.31 | 48.38 | 6.30 | 8.37 |
| Gulmi | Chandrakot | 50403 | 56.13 | 48.24 | 6.29 | 8.36 |
| Gulmi | Musikot | 50404 | 54.78 | 47.15 | 6.39 | 8.44 |
| Gulmi | Isma | 50405 | 57.14 | 49.04 | 6.39 | 8.44 |
| Gulmi | Malika | 50406 | 54.69 | 47.34 | 6.29 | 8.62 |
| Gulmi | Madane | 50407 | 55.95 | 48.35 | 6.28 | 8.61 |
| Gulmi | Dhurkot | 50408 | 55.81 | 48.24 | 6.39 | 8.70 |
| Gulmi | Resunga | 50409 | 54.86 | 47.22 | 6.39 | 8.44 |
| Gulmi | Gulmidarbar | 50410 | 52.45 | 45.29 | 6.39 | 8.44 |
| Gulmi | Chatrakot | 50411 | 55.03 | 47.35 | 6.29 | 8.36 |
| Gulmi | Ruru | 50412 | 56.65 | 48.65 | 6.46 | 8.50 |
| Arghakhanchi | Chhatradev | 50501 | 53.91 | 46.82 | 5.39 | 8.01 |
| Arghakhanchi | Malarani | 50502 | 56.14 | 48.61 | 5.32 | 7.95 |
| Arghakhanchi | Bhumekasthan | 50503 | 60.59 | 53.28 | 5.32 | 9.06 |
| Arghakhanchi | Sandhikharka | 50504 | 60.06 | 52.85 | 5.32 | 9.06 |
| Arghakhanchi | Panini | 50505 | 60.06 | 52.85 | 5.32 | 9.06 |
| Arghakhanchi | Sitganga | 50506 | 61.58 | 54.07 | 5.35 | 9.08 |
| Palpa | Rampur | 50601 | 56.37 | 50.30 | 6.30 | 10.25 |
| Palpa | Purbakhola | 50602 | 58.76 | 52.21 | 6.29 | 10.24 |
| Palpa | Rambha | 50603 | 56.37 | 50.30 | 6.30 | 10.25 |
| Palpa | Bagnaskali | 50604 | 56.65 | 50.05 | 6.46 | 9.89 |
| Palpa | Tansen | 50605 | 58.89 | 51.83 | 6.46 | 9.89 |
| Palpa | Ribdikot | 50606 | 56.65 | 50.32 | 6.46 | 10.16 |
| Palpa | Rainadevi Chhahara | 50607 | 62.41 | 56.29 | 6.35 | 11.44 |
| Palpa | Tinau | 50608 | 63.80 | 56.04 | 7.37 | 10.89 |
| Palpa | Mathagadhi | 50609 | 62.77 | 55.42 | 6.46 | 10.37 |
| Palpa | Nisdi | 50610 | 59.88 | 53.11 | 6.29 | 10.24 |
| Nawalparasi_W | Bardaghat | 50701 | 60.07 | 51.28 | 5.58 | 7.70 |
| Nawalparasi_W | Sunwal | 50702 | 61.79 | 52.66 | 5.63 | 7.74 |
| Nawalparasi_W | Ramgram | 50703 | 61.95 | 52.79 | 5.79 | 7.87 |
| Nawalparasi_W | Palhi Nandan | 50704 | 59.75 | 50.87 | 5.79 | 7.71 |
| Nawalparasi_W | Sarawal | 50705 | 55.74 | 47.82 | 5.79 | 7.87 |
| Nawalparasi_W | Pratappur | 50706 | 57.86 | 49.36 | 5.58 | 7.54 |
| Nawalparasi_W | Susta | 50707 | 57.86 | 49.52 | 5.58 | 7.70 |
| Rupandehi | Devdaha | 50801 | 66.13 | 59.04 | 9.37 | 13.62 |
| Rupandehi | Butwal | 50802 | 68.15 | 60.92 | 9.37 | 13.90 |
| Rupandehi | Sainamaina | 50803 | 65.88 | 60.48 | 9.37 | 15.27 |
| Rupandehi | Kanchan | 50804 | 64.29 | 57.84 | 8.57 | 13.26 |
| Rupandehi | Gaidahawa | 50805 | 64.38 | 57.91 | 8.65 | 13.33 |
| Rupandehi | Sudhdhodhan | 50806 | 66.13 | 59.31 | 9.37 | 13.90 |
| Rupandehi | Siyari | 50807 | 66.27 | 58.99 | 8.79 | 13.01 |
| Rupandehi | Tillotama | 50808 | 66.13 | 59.04 | 9.37 | 13.62 |
| Rupandehi | Omsatiya | 50809 | 65.16 | 58.26 | 9.76 | 13.94 |
| Rupandehi | Rohini | 50810 | 64.26 | 57.38 | 9.76 | 13.78 |
| Rupandehi | Siddharthanagar | 50811 | 67.13 | 59.68 | 10.76 | 14.58 |
| Rupandehi | Mayadevi | 50812 | 65.84 | 58.91 | 9.76 | 14.05 |
| Rupandehi | Lumbini Sanskritik | 50813 | 60.27 | 54.46 | 8.75 | 13.24 |
| Rupandehi | Kotahimai | 50814 | 63.03 | 56.66 | 8.80 | 13.28 |
| Rupandehi | Sammarimai | 50815 | 59.11 | 53.53 | 8.80 | 13.28 |
| Rupandehi | Marchawari | 50816 | 60.07 | 53.44 | 8.80 | 12.43 |
| Kapilbastu | Banganga | 50901 | 61.48 | 54.32 | 7.57 | 11.20 |
| Kapilbastu | Buddhabhumi | 50902 | 61.47 | 54.32 | 7.56 | 11.19 |
| Kapilbastu | Shivaraj | 50903 | 61.82 | 54.60 | 7.65 | 11.26 |
| Kapilbastu | Bijayanagar | 50904 | 59.65 | 52.86 | 7.65 | 11.26 |
| Kapilbastu | Krishnanagar | 50905 | 59.67 | 52.88 | 7.65 | 11.26 |
| Kapilbastu | Maharajgunj | 50906 | 61.57 | 54.40 | 7.65 | 11.26 |
| Kapilbastu | Kapilbastu | 50907 | 63.64 | 54.69 | 7.58 | 9.84 |
| Kapilbastu | Yashodhara | 50908 | 61.50 | 52.81 | 7.58 | 9.67 |
| Kapilbastu | Mayadevi | 50909 | 61.50 | 52.98 | 7.58 | 9.84 |
| Kapilbastu | Suddhodhan | 50910 | 61.60 | 53.06 | 7.65 | 9.89 |
| Dang | Banglachuli | 51001 | 59.72 | 52.86 | 8.30 | 11.72 |
| Dang | Ghorahi | 51002 | 62.08 | 54.74 | 9.37 | 12.57 |
| Dang | Tulsipur | 51003 | 64.07 | 56.34 | 9.37 | 12.57 |
| Dang | Shantinagar | 51004 | 60.91 | 53.81 | 8.37 | 11.77 |
| Dang | Babai | 51005 | 65.63 | 57.58 | 8.22 | 11.65 |
| Dang | Dangisharan | 51006 | 63.92 | 56.21 | 8.37 | 11.77 |
| Dang | Lamahi | 51007 | 63.72 | 56.06 | 8.37 | 11.77 |
| Dang | Rapti | 51008 | 63.66 | 55.79 | 8.25 | 11.46 |
| Dang | Gadhawa | 51009 | 62.58 | 54.93 | 8.25 | 11.46 |
| Dang | Rajpur | 51010 | 65.19 | 57.23 | 8.15 | 11.60 |
| Banke | Rapti Sonari | 51101 | 67.24 | 59.17 | 8.41 | 12.11 |
| Banke | Kohalpur | 51102 | 66.54 | 58.61 | 9.61 | 13.07 |
| Banke | Baijanath | 51103 | 62.27 | 55.19 | 8.50 | 12.18 |
| Banke | Khajura | 51104 | 67.75 | 59.58 | 8.60 | 12.25 |
| Banke | Janki | 51105 | 66.74 | 58.77 | 9.61 | 13.07 |
| Banke | Nepalgunj | 51106 | 69.56 | 61.02 | 10.61 | 13.87 |
| Banke | Duduwa | 51107 | 66.54 | 58.61 | 9.61 | 13.07 |
| Banke | Narainapur | 51108 | 65.09 | 57.45 | 8.41 | 12.11 |
| Bardiya | Bansagadhi | 51201 | 59.42 | 52.86 | 6.41 | 10.46 |
| Bardiya | Barbardiya | 51202 | 57.44 | 51.28 | 6.56 | 10.58 |
| Bardiya | Thakurbaba | 51203 | 59.47 | 52.35 | 6.42 | 9.92 |
| Bardiya | Geruwa | 51204 | 56.30 | 49.81 | 6.68 | 10.12 |
| Bardiya | Rajapur | 51205 | 56.30 | 48.14 | 6.68 | 8.45 |
| Bardiya | Madhuwan | 51206 | 57.44 | 50.73 | 6.56 | 10.03 |
| Bardiya | Gulariya | 51207 | 61.74 | 52.58 | 6.56 | 8.45 |
| Bardiya | Badhaiyatal | 51208 | 59.57 | 52.98 | 6.56 | 10.58 |
| Dolpa | Dolpo Buddha | 60101 | 13.95 | 15.55 | 5.01 | 8.40 |
| Dolpa | Shey Phoksundo | 60102 | 47.57 | 42.50 | 5.03 | 8.47 |
| Dolpa | Jagadulla | 60103 | 52.48 | 46.74 | 5.03 | 8.77 |
| Dolpa | Mudkechula | 60104 | 51.35 | 45.83 | 5.07 | 8.80 |
| Dolpa | Tripurasundari | 60105 | 49.31 | 44.20 | 5.06 | 8.80 |
| Dolpa | Thuli Bheri | 60106 | 51.81 | 46.20 | 5.06 | 8.80 |
| Dolpa | Kaike | 60107 | 13.51 | 15.20 | 5.02 | 8.41 |
| Dolpa | Chharka Tangsong | 60108 | 13.28 | 15.02 | 5.02 | 8.40 |
| Mugu | Mugum Karmarong | 60201 | 46.23 | 41.51 | 5.04 | 8.56 |
| Mugu | Chhayanath Rara | 60202 | 50.94 | 45.56 | 5.06 | 8.85 |
| Mugu | Soru | 60203 | 48.05 | 43.24 | 5.06 | 8.85 |
| Mugu | Khatyad | 60204 | 53.89 | 47.92 | 5.12 | 8.90 |
| Humla | Chankheli | 60301 | 11.57 | 13.86 | 5.04 | 8.64 |
| Humla | Kharpunath | 60302 | 48.58 | 43.47 | 5.03 | 8.63 |
| Humla | Simkot | 60303 | 48.57 | 43.46 | 5.01 | 8.62 |
| Humla | Namkha | 60304 | 52.36 | 46.50 | 5.01 | 8.62 |
| Humla | Sarkegad | 60305 | 48.54 | 43.44 | 5.05 | 8.64 |
| Humla | Adanchuli | 60306 | 11.67 | 13.95 | 5.05 | 8.64 |
| Humla | Tanjakot | 60307 | 48.20 | 43.45 | 5.06 | 8.94 |
| Jumla | Patrasi | 60401 | 54.16 | 48.32 | 7.19 | 10.74 |
| Jumla | Kanakasundari | 60402 | 56.38 | 50.07 | 7.19 | 10.72 |
| Jumla | Sinja | 60403 | 58.96 | 52.16 | 7.19 | 10.74 |
| Jumla | Chandannath | 60404 | 59.65 | 52.71 | 7.19 | 10.74 |
| Jumla | Guthichaur | 60405 | 57.15 | 50.71 | 7.19 | 10.74 |
| Jumla | Tatopani | 60406 | 55.83 | 49.66 | 7.19 | 10.75 |
| Jumla | Tila | 60407 | 59.25 | 52.40 | 7.08 | 10.66 |
| Jumla | Hima | 60408 | 61.80 | 54.43 | 7.08 | 10.66 |
| Kalikot | Palata | 60501 | 56.69 | 50.32 | 5.12 | 9.06 |
| Kalikot | Pachaljharana | 60502 | 50.73 | 45.55 | 5.27 | 9.19 |
| Kalikot | Raskot | 60503 | 55.77 | 49.58 | 5.27 | 9.19 |
| Kalikot | Sanni Tribeni | 60504 | 52.80 | 47.39 | 5.27 | 9.36 |
| Kalikot | Naraharinath | 60505 | 53.13 | 47.65 | 5.19 | 9.29 |
| Kalikot | Khandachakra | 60506 | 50.74 | 45.74 | 5.17 | 9.29 |
| Kalikot | Tilagufa | 60507 | 53.50 | 47.80 | 5.15 | 9.12 |
| Kalikot | Mahawai | 60508 | 50.94 | 45.76 | 5.19 | 9.15 |
| Kalikot | Kalika | 60509 | 53.16 | 47.67 | 5.21 | 9.32 |
| Dailekh | Naumule | 60601 | 61.41 | 54.54 | 5.24 | 9.60 |
| Dailekh | Mahabu | 60602 | 58.61 | 52.30 | 5.24 | 9.60 |
| Dailekh | Bhairabi | 60603 | 57.79 | 51.79 | 5.29 | 9.78 |
| Dailekh | Thantikandh | 60604 | 50.68 | 46.09 | 5.29 | 9.78 |
| Dailekh | Aathabis | 60605 | 61.27 | 54.57 | 5.29 | 9.78 |
| Dailekh | Chamunda Bindrasaini | 60606 | 60.46 | 53.92 | 5.29 | 9.78 |
| Dailekh | Dullu | 60607 | 62.74 | 55.74 | 5.29 | 9.78 |
| Dailekh | Narayan | 60608 | 60.43 | 53.75 | 5.26 | 9.62 |
| Dailekh | Bhagawatimai | 60609 | 63.49 | 56.35 | 5.24 | 9.75 |
| Dailekh | Dungeshwor | 60610 | 62.72 | 55.73 | 5.26 | 9.76 |
| Dailekh | Gurans | 60611 | 63.30 | 56.20 | 5.41 | 9.88 |
| Jajarkot | Barekot | 60701 | 47.84 | 43.33 | 5.08 | 9.12 |
| Jajarkot | Kuse | 60702 | 58.36 | 51.75 | 5.15 | 9.18 |
| Jajarkot | Junichande | 60703 | 60.66 | 53.59 | 5.12 | 9.17 |
| Jajarkot | Chhedagad | 60704 | 60.64 | 53.58 | 5.15 | 9.19 |
| Jajarkot | Shiwalaya | 60705 | 63.37 | 55.76 | 5.12 | 9.17 |
| Jajarkot | Bheri | 60706 | 59.19 | 52.42 | 5.26 | 9.27 |
| Jajarkot | Tribeni Nalagad | 60707 | 54.18 | 48.40 | 5.15 | 9.18 |
| Rukum_W | Aathbiskot | 60801 | 52.48 | 47.04 | 5.17 | 9.19 |
| Rukum_W | Sani Bheri | 60802 | 51.01 | 45.86 | 5.26 | 9.26 |
| Rukum_W | Banfikot | 60803 | 50.99 | 45.85 | 5.24 | 9.25 |
| Rukum_W | Musikot | 60804 | 53.75 | 48.06 | 5.24 | 9.25 |
| Rukum_W | Tribeni | 60805 | 54.46 | 48.63 | 5.26 | 9.26 |
| Rukum_W | Chaurjahari | 60806 | 57.71 | 51.22 | 5.26 | 9.26 |
| Salyan | Darma | 60901 | 54.12 | 48.71 | 5.26 | 9.62 |
| Salyan | Kumakhmalika | 60902 | 56.35 | 50.50 | 5.26 | 9.62 |
| Salyan | Bangad Kupinde | 60903 | 60.24 | 53.61 | 5.19 | 9.57 |
| Salyan | Dhorchaur | 60904 | 53.60 | 48.30 | 5.21 | 9.58 |
| Salyan | Bagchaur | 60905 | 54.50 | 49.02 | 5.24 | 9.61 |
| Salyan | Chhatreshwori | 60906 | 54.46 | 48.65 | 5.21 | 9.24 |
| Salyan | Sharada | 60907 | 56.31 | 50.46 | 5.21 | 9.58 |
| Salyan | Kalimati | 60908 | 62.05 | 55.06 | 5.38 | 9.72 |
| Salyan | Tribeni | 60909 | 58.25 | 51.67 | 5.37 | 9.37 |
| Salyan | Kapurkot | 60910 | 57.91 | 51.40 | 5.37 | 9.37 |
| Surkhet | Simta | 61001 | 66.27 | 58.83 | 8.19 | 12.37 |
| Surkhet | Chingad | 61002 | 66.65 | 59.28 | 8.41 | 12.69 |
| Surkhet | Lekbeshi | 61003 | 66.65 | 59.13 | 8.41 | 12.54 |
| Surkhet | Gurbhakot | 61004 | 66.27 | 58.83 | 8.19 | 12.37 |
| Surkhet | Bheriganga | 61005 | 66.65 | 59.28 | 8.41 | 12.69 |
| Surkhet | Birendranagar | 61006 | 69.73 | 61.75 | 9.41 | 13.49 |
| Surkhet | Barahtal | 61007 | 66.65 | 59.28 | 8.41 | 12.69 |
| Surkhet | Panchpuri | 61008 | 63.58 | 56.83 | 8.11 | 12.45 |
| Surkhet | Chaukune | 61009 | 63.58 | 56.83 | 8.11 | 12.45 |
| Bajura | Himali | 70101 | 51.74 | 46.44 | 5.12 | 9.14 |
| Bajura | Gaumul | 70102 | 53.11 | 47.72 | 5.15 | 9.34 |
| Bajura | Budhinanda | 70103 | 50.63 | 45.56 | 5.08 | 9.11 |
| Bajura | Swami Kartik | 70104 | 53.74 | 48.04 | 5.12 | 9.14 |
| Bajura | Pandav Gupha | 70105 | 47.06 | 42.88 | 5.27 | 9.45 |
| Bajura | Badimalika | 70106 | 47.04 | 42.86 | 5.37 | 9.52 |
| Bajura | Chhededaha | 70107 | 57.58 | 51.29 | 5.37 | 9.52 |
| Bajura | Budhiganga | 70108 | 56.36 | 50.32 | 5.37 | 9.52 |
| Bajura | Tribeni | 70109 | 52.96 | 47.59 | 5.37 | 9.52 |
| Bajhang | Kanda | 70201 | 52.37 | 46.74 | 5.07 | 8.91 |
| Bajhang | Bungal | 70202 | 56.24 | 49.84 | 5.21 | 9.02 |
| Bajhang | Surma | 70203 | 55.25 | 49.05 | 5.14 | 8.96 |
| Bajhang | Talkot | 70204 | 55.97 | 49.62 | 5.14 | 8.96 |
| Bajhang | Masta | 70205 | 55.97 | 49.49 | 5.14 | 8.83 |
| Bajhang | JayaPrithivi | 70206 | 58.42 | 51.45 | 5.14 | 8.83 |
| Bajhang | Chabispathivera | 70207 | 55.32 | 49.03 | 5.21 | 8.94 |
| Bajhang | Durgathali | 70208 | 58.88 | 51.87 | 5.21 | 8.94 |
| Bajhang | Kedarseu | 70209 | 56.54 | 50.00 | 5.21 | 8.94 |
| Bajhang | Bithadchir | 70210 | 53.66 | 47.70 | 5.20 | 8.93 |
| Bajhang | Thalara | 70211 | 52.18 | 46.51 | 5.21 | 8.94 |
| Bajhang | Khaptadchhanna | 70212 | 56.00 | 50.11 | 5.17 | 9.45 |
| Darchula | Byas | 70301 | 55.93 | 49.56 | 5.17 | 8.95 |
| Darchula | Dunhu | 70302 | 54.57 | 48.34 | 5.17 | 8.82 |
| Darchula | Mahakali | 70303 | 59.43 | 51.80 | 5.19 | 8.40 |
| Darchula | Naugad | 70304 | 57.38 | 50.59 | 5.19 | 8.84 |
| Darchula | Apihimal | 70305 | 53.76 | 47.83 | 5.09 | 8.89 |
| Darchula | Marma | 70306 | 54.64 | 48.40 | 5.19 | 8.84 |
| Darchula | Shailyashikhar | 70307 | 57.31 | 50.53 | 5.19 | 8.84 |
| Darchula | Malikaarjun | 70308 | 54.52 | 47.87 | 5.19 | 8.40 |
| Darchula | Lekam | 70309 | 54.26 | 48.09 | 5.26 | 8.89 |
| Baitadi | Dilasaini | 70401 | 54.39 | 48.28 | 5.20 | 8.92 |
| Baitadi | Dogadakedar | 70402 | 54.06 | 48.02 | 5.26 | 8.97 |
| Baitadi | Purchaudi | 70403 | 54.39 | 48.33 | 5.20 | 8.98 |
| Baitadi | Surnaya | 70404 | 52.95 | 47.18 | 5.26 | 9.02 |
| Baitadi | Dasharathchanda | 70405 | 51.87 | 45.82 | 5.26 | 8.54 |
| Baitadi | Pancheshwar | 70406 | 51.38 | 45.44 | 5.26 | 8.54 |
| Baitadi | Shivanath | 70407 | 49.04 | 43.57 | 5.21 | 8.50 |
| Baitadi | Melauli | 70408 | 52.58 | 46.40 | 5.26 | 8.54 |
| Baitadi | Patan | 70409 | 55.90 | 49.54 | 5.26 | 9.02 |
| Baitadi | Sigas | 70410 | 53.27 | 47.44 | 5.20 | 8.98 |
| Dadeldhura | Nawadurga | 70501 | 53.63 | 48.04 | 5.19 | 9.28 |
| Dadeldhura | Amargadhi | 70502 | 59.21 | 52.50 | 5.15 | 9.25 |
| Dadeldhura | Ajaymeru | 70503 | 53.09 | 47.11 | 5.19 | 8.79 |
| Dadeldhura | Bhageshwar | 70504 | 51.32 | 46.19 | 5.21 | 9.30 |
| Dadeldhura | Parashuram | 70505 | 59.85 | 53.01 | 5.61 | 9.62 |
| Dadeldhura | Alital | 70506 | 57.58 | 51.20 | 5.25 | 9.33 |
| Dadeldhura | Ganayapdhura | 70507 | 51.32 | 46.19 | 5.15 | 9.25 |
| Doti | Purbichauki | 70601 | 56.68 | 50.74 | 5.26 | 9.60 |
| Doti | Sayal | 70602 | 54.73 | 49.18 | 5.26 | 9.60 |
| Doti | Adharsha | 70603 | 58.25 | 52.00 | 5.26 | 9.60 |
| Doti | Shikhar | 70604 | 56.68 | 50.74 | 5.26 | 9.60 |
| Doti | Dipayal Silgadi | 70605 | 56.46 | 50.56 | 5.26 | 9.60 |
| Doti | K I Singh | 70606 | 59.19 | 52.75 | 5.26 | 9.60 |
| Doti | Bogtan | 70607 | 59.85 | 53.27 | 5.16 | 9.53 |
| Doti | Badikedar | 70608 | 59.89 | 53.30 | 5.16 | 9.53 |
| Doti | Jorayal | 70609 | 56.77 | 50.27 | 5.11 | 8.95 |
| Achham | Panchadewal Binayak | 70701 | 53.54 | 48.83 | 5.20 | 10.15 |
| Achham | Ramaroshan | 70702 | 50.75 | 46.59 | 5.30 | 10.23 |
| Achham | Mellekh | 70703 | 56.75 | 51.40 | 5.37 | 10.29 |
| Achham | Sanphebagar | 70704 | 59.31 | 53.44 | 5.37 | 10.29 |
| Achham | Chaurpati | 70705 | 56.42 | 51.13 | 5.26 | 10.20 |
| Achham | Mangalsen | 70706 | 56.88 | 51.50 | 5.30 | 10.23 |
| Achham | Bannigadhi Jayagadh | 70707 | 56.30 | 51.03 | 5.30 | 10.23 |
| Achham | Kamalbazar | 70708 | 60.93 | 54.74 | 5.20 | 10.15 |
| Achham | Dhakari | 70709 | 61.33 | 55.06 | 5.20 | 10.15 |
| Achham | Turmakhad | 70710 | 66.12 | 58.89 | 8.29 | 12.63 |
| Kailali | Mohanyal | 70801 | 63.87 | 57.71 | 8.42 | 13.35 |
| Kailali | Chure | 70802 | 63.29 | 56.71 | 8.25 | 12.68 |
| Kailali | Godawari | 70803 | 64.50 | 57.68 | 8.56 | 12.93 |
| Kailali | Gauriganga | 70804 | 64.37 | 57.58 | 8.56 | 12.93 |
| Kailali | Ghodaghodi | 70805 | 63.66 | 57.01 | 8.55 | 12.92 |
| Kailali | Bardagoriya | 70806 | 61.51 | 55.29 | 8.55 | 12.92 |
| Kailali | Lamkichuha | 70807 | 61.51 | 55.29 | 8.55 | 12.92 |
| Kailali | Janaki | 70808 | 60.07 | 53.63 | 8.68 | 12.52 |
| Kailali | Joshipur | 70809 | 61.84 | 53.37 | 8.68 | 10.84 |
| Kailali | Tikapur | 70810 | 60.07 | 51.96 | 8.68 | 10.84 |
| Kailali | Bhajani | 70811 | 63.97 | 55.08 | 8.68 | 10.84 |
| Kailali | Kailari | 70812 | 64.50 | 55.50 | 8.56 | 10.75 |
| Kailali | Dhangadhi | 70813 | 67.51 | 59.29 | 9.56 | 12.93 |
| Kanchanpur | Krishnapur | 70901 | 63.17 | 56.01 | 7.56 | 11.52 |
| Kanchanpur | Shuklaphanta | 70902 | 62.42 | 54.61 | 7.29 | 10.50 |
| Kanchanpur | Bedkot | 70903 | 59.43 | 52.22 | 7.61 | 10.76 |
| Kanchanpur | Bhimdatta | 70904 | 59.51 | 52.28 | 7.69 | 10.82 |
| Kanchanpur | Mahakali | 70905 | 57.52 | 50.69 | 7.69 | 10.82 |
| Kanchanpur | Laljhadi | 70906 | 62.64 | 54.79 | 7.52 | 10.69 |
| Kanchanpur | Punarbas | 70907 | 63.17 | 55.21 | 7.56 | 10.72 |
| Kanchanpur | Belauri | 70908 | 62.84 | 54.95 | 7.60 | 10.75 |
| Kanchanpur | Beldandi | 70909 | 55.90 | 49.40 | 7.60 | 10.75 |

Table A-10 Relative percentage of patients with different symptoms

| Source | Guan et al^6^ | Zhou et al^7^ | Garg et al^8^ | Average |
| --- | --- | --- | --- | --- |
| Country | China | China (Wuhan) | USA |  |
| Sample | 1099 | 191 | 180(Max) |  |
| Fever | 100% | 100% | 99% | 100% |
| Nasal congestion | 5% |  | 19% | 12% |
| Headache | 15% |  | 19% | 17% |
| cough | 76% | 84% | 100% | 87% |
| Sore throat | 16% |  | 21% | 18% |
| Sputum production | 38% | 24% |  | 31% |
| Fatigue | 43% | 24% |  | 34% |
| Shortness of breathe | 21% |  | 93% | 57% |
| Nausea or vomiting | 6% | 4% | 28% | 13% |
| Diarrhea | 4% | 5% | 31% | 14% |
| Myalgia | 17% | 16% | 40% | 24% |
| chills | 13% |  |  | 13% |
| Chest pain |  |  | 17% | 17% |


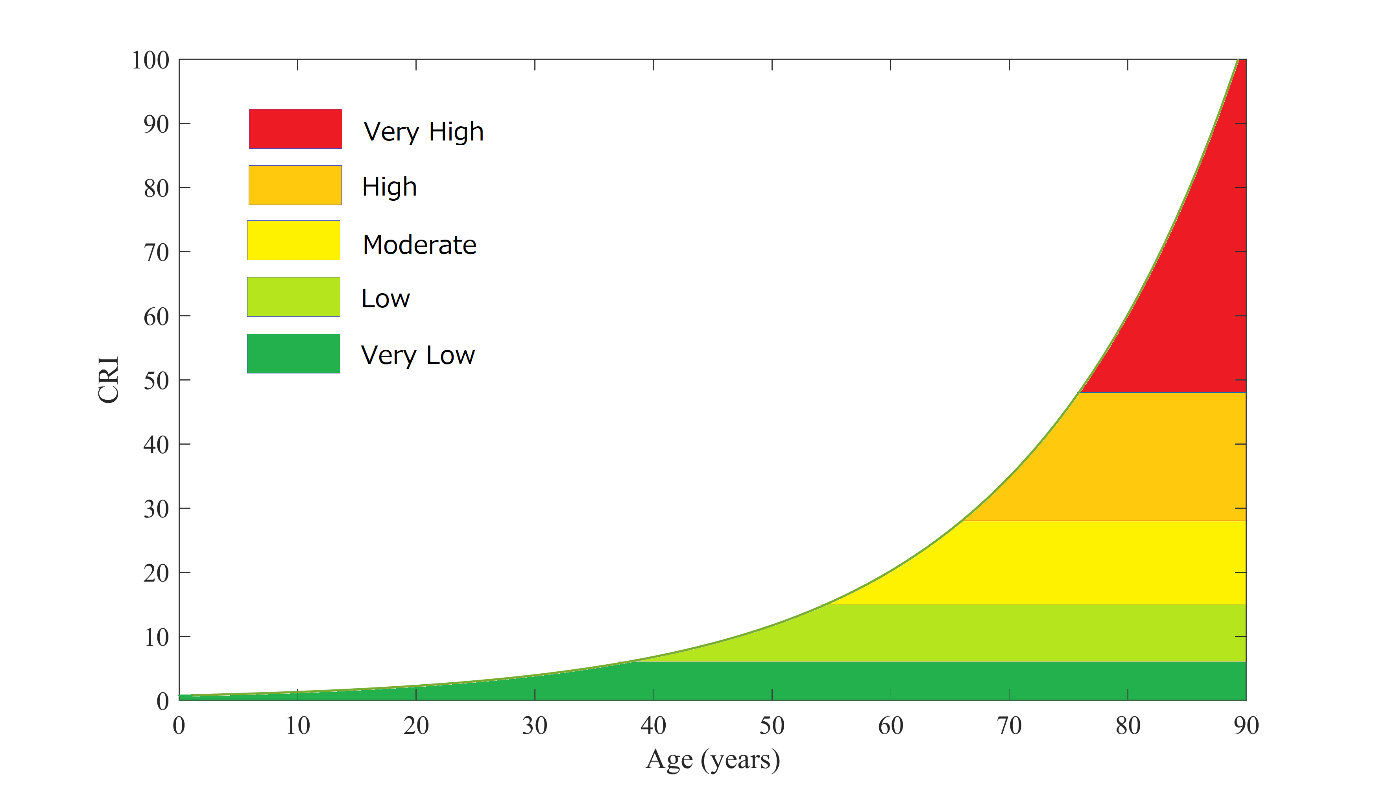


Figure A-1 Risk level stratification considering equal risk area distribution over ranges
